# Synthesis of Near-Infrared-Absorbing Anionic Heptamethine Cyanine Dyes with Trifluoromethyl Groups

**DOI:** 10.3390/molecules28124650

**Published:** 2023-06-08

**Authors:** Hiroki Masuoka, Yasuhiro Kubota, Toshiyasu Inuzuka, Kazumasa Funabiki

**Affiliations:** 1 Department of Chemistry and Biomolecular Science, Gifu University, 1-1 Yanagido, Gifu 501-1193, Japan; 2 Division of Instrumental Analysis, Life Science Research Center, Gifu University, 1-1 Yanagido, Gifu 501-1193, Japan

**Keywords:** heptamethine cyanine dye, near-infrared, trifluoromethyl

## Abstract

A novel anionic heptamethine cyanine (HMC) dye with two trifluoromethyl groups that selectively absorb near-infrared light is synthesized. When contrasted with previously studied anionic HMC dyes with substituents such as methyl, phenyl, and pentafluorophenyl groups, the trifluoromethylated dye displays a red-shifted maximum absorption wavelength (for instance, 948 nm in CH_2_Cl_2_) along with enhanced photostability. Furthermore, HMC dyes with broad absorption in the near-infrared region are synthesized by combining a trifluoromethylated anionic HMC dye with a cationic HMC dye as a counterion.

## 1. Introduction

**Figure 1:**
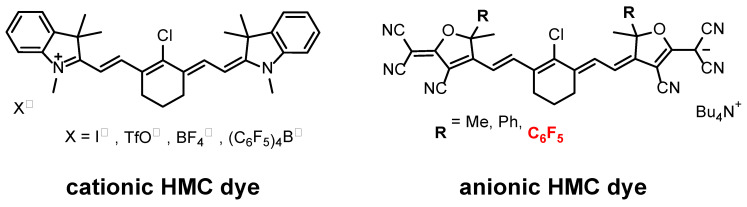
Structures of HMC dyes.

Near-infrared (NIR)-absorbing organic dyes have garnered considerable attention for their potential applications in biology [1,2,3], transparent solar cells [4,5,6,7,8,9,10,11,12,13,14,15], optical sensors [16,17,18], and optical communications [19,20,21]. These dyes are particularly important as they allow us to take advantage of NIR light, a resource that has yet to be fully exploited. Among them, heptamethine cyanine (HMC) dye [22,23,24,25,26,27,28] is an organic dye that absorbs NIR light with excellent optical properties, such as selective absorption in the NIR region and a high molar absorption coefficient. Figure 1 illustrates the two categories of HMC dyes—cationic and anionic. Notably, extensive literature is available detailing the synthesis and various applications of cationic HMC dyes [29,30,31,32]. However, reports on anionic HMC dyes are limited [33,34,35]. Generally, anionic HMC dyes exhibit superior optical properties, including a red-shifted maximum absorption wavelength (λ_max_) relative to their cationic counterparts. However, comparatively lower photostability hinders their application [36,37].

We previously reported that introducing perfluorophenyl groups into an anionic HMC dye resulted in more red-shifted λ_max_ and higher photostability of the dye than those with methyl or phenyl groups [37]. Since the mechanism of photolysis of HMC dyes is based on the addition of an electrophilic singlet oxygen to a double bond, the introduction of electron-withdrawing properties into the HMC dye backbone should be the most powerful means of improving its photostability. Among them, the introduction of the CF_3_ group, one of the most electron-withdrawing substituents, can be expected to be an effective means of improving the photostability of the dye. Herein, we report the synthesis and optical properties of novel anionic HMC-dye-bearing trifluoromethyl groups. Specifically, the trifluoromethylated dye achieved a more red-shifted λ_max_, lower highest occupied molecular orbital (HOMO) level, and higher photostability than the previously reported anionic HMC dyes.

On the other hand, the synthesis of cyanine–cyanine mixed dyes consisting of a cationic HMC skeleton and an anionic HMC skeleton to broaden the narrow absorption range of HMC dyes is an excellent method, but the optical properties and applications of cyanine–cyanine mixed dyes composed of cationic and anionic HMC skeletons to photoelectric conversion devices have been scarcely reported recently [38,39,40]. Additionally, their photostability has not been investigated so far. Therefore, a novel mixture of trifluoromethylated anionic HMC dye and cationic HMC dye was also synthesized and its absorption properties and photostability were investigated.

## 2. Results

### 2.1. Synthesis of the Anionic Dye with Trifluoromethyl Groups ***5a*** and ***8a***

**Scheme 1:**
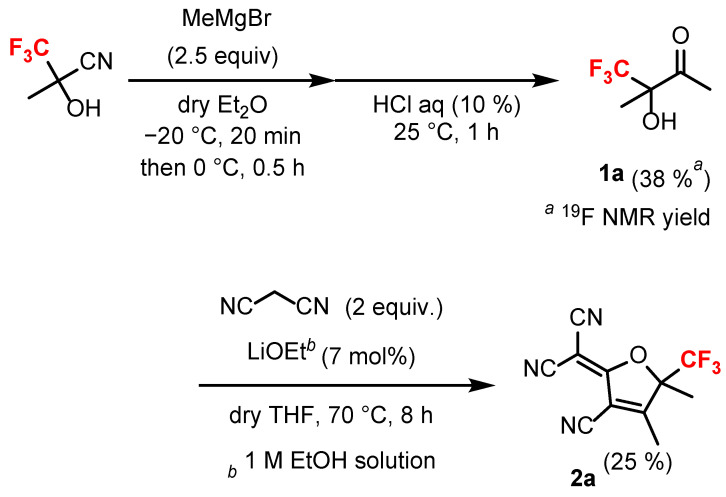
Synthesis of trifluoromethylated tricyanofuran **2a**.

Anionic HMC dyes with trifluoromethyl groups were prepared as follows. First, tricyanofurans bearing trifluoromethyl groups were synthesized (Scheme 1). 2-Hydroxy-2-(trifluoromethyl)propionitrile and methylmagnesium bromide were treated in dehydrated diethyl ether (Et_2_O) at −20 °C. The temperature was gradually increased to 0 °C and stirred for 30 min. The reaction was quenched with 10% HCl and stirred at 25 °C for 1 h, resulting in the production of 4,4,4-trifluoro-3-hydroxy-3-methylbutan-2-one (**1a**), which returned a 38% yield as determined by ^19^F nuclear magnetic resonance (NMR) analysis [41]. A couple of reasons for the low yield of **1a** may be due to the reaction not progressing sufficiently and the high volatility of **1a** when the extraction solvent was removed. The ketone **1a** was used in the following reaction without further purification. A mixture of **1a** and 2 equiv. of malononitrile and a catalytic amount of lithium ethoxide in dehydrated THF was stirred under reflux for 8 h to obtain 2-(2-cyano-3,4-dimethyl-4-(trifluoromethyl)cyclopent-2-en-1-ylidene)malononitrile **2a** at 25% yield [42]. In the synthesis of various tricyanofurans, it has been reported that only low yields can be obtained when hydroxyketones with electron-withdrawing substituents are used [43]. In the present study, the extremely strong electron-withdrawing CF_3_ group seems to have a significant influence on the low yield of tricyanofuran **2a**.

**Scheme 2:**
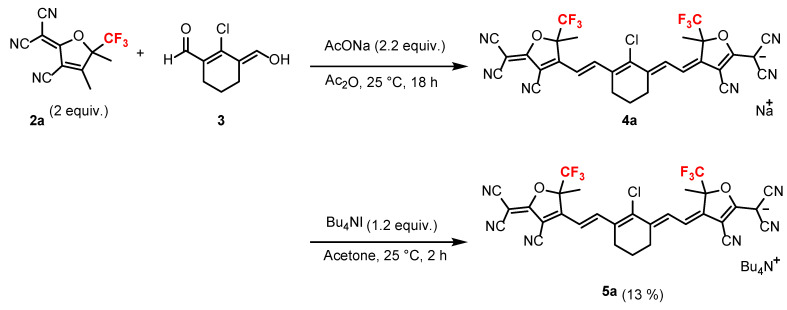
Synthesis of the trifluoromethylated anionic HMC dye **5a**.

The sodium salt of anionic HMC dye **4a** was obtained via the reaction of dialdehyde **3** [44,45] with two equiv. of trifluoromethylated tricyanofuran **2a** in sodium acetate in anhydrous acetic acid at 25 °C overnight (Scheme 2). The crude sodium salt of the HMC dye **4a** was used in subsequent reactions without further purification. A mixture of crude **4a** and tetrabutylammonium iodide (Bu_4_N^+^I^−^) was stirred at 25 °C to afford the trifluoromethylated anionic HMC dye **5a** with tetrabutylammonium cation. The overall yield of tricyanofuran **2a** to trifluoromethylated anionic HMC dye **5a** was 13% from 3. The low yield may be due to the reaction to synthesize the sodium salt **4a** not progressing sufficiently.

**Scheme 3:**
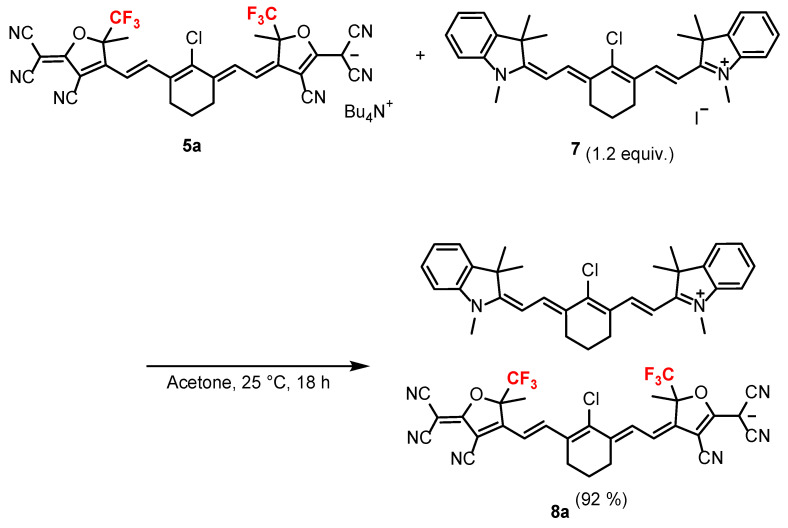
Synthesis of the cyanine–cyanine mixed dye **8a**.

The tetrabutylammonium salt of the anionic HMC dye **5a** was stirred with 1.2 equiv. of the cationic HMC dye **7** [46], which was prepared via the reaction of indolenium salt **6 [47]** with dialdehyde **3** in acetone overnight at 25 °C to afford the cyanine–cyanine mixed dye **8a** with a yield of 92% (Scheme 3).

### 2.2. UV–vis–NIR Spectra and CV Measurements of Anionic HMC Dye ***5a*** and Cyanine–Cyanine Mixed Dye ***8a***

**Figure 2:**
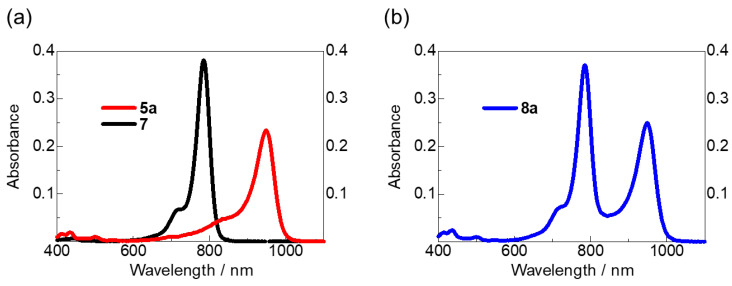
UV–vis–NIR absorption spectra of the prepared anionic HMC dye **5a** and cationic HMC dye **7** (**a**), and cyanine–cyanine dye **8a** (**b**) in CH_2_Cl_2_ (1 × 10^−6^ M).

The ultraviolet–visible–NIR (UV–vis–NIR) absorption spectra of the prepared trifluoromethylated anionic HMC dye **5a** with Bu_4_N^+^ cations and cyanine–cyanine mixed dye **8a** in a dichloromethane (CH_2_Cl_2_) solution are shown in Figure 2a for the prepared trifluoromethylated anionic HMC dye **5a** and cationic HMC dye. Figure 2b shows the cyanine–cyanine mixed dye **8a**. Table 1 summarizes λ_max_, molar absorption coefficient (*ε*), oxidation potential (*E*_ox_), HOMO, and lowest unoccupied molecular orbital (LUMO) levels of the previously synthesized anionic HMC dyes.

As a result, the λ_max_ of the dye **5a** was observed at 948 nm, with negligible absorption in the visible region. However, cyanine–cyanine mixed dye **8a** showed absorption from the cationic and anionic HMC skeletons at 785 and 949 nm, respectively, giving a broader absorption range than that of anionic HMC dye **5a**. Compared with the various anionic HMC dyes synthesized, **5a** showed a significant red shift in λ_max_, stabilization of each energy level, and decreased HOMO−LUMO energy gap. These results can be attributed to the trifluoromethyl groups being stronger electron-withdrawing substituents than the substituents of other anionic HMC dyes.

### 2.3. The Photostabilities of Anionic HMC Dye ***5a*** and Cyanine−Cyanine Mixed Dye ***8a***

**Figure 3:**
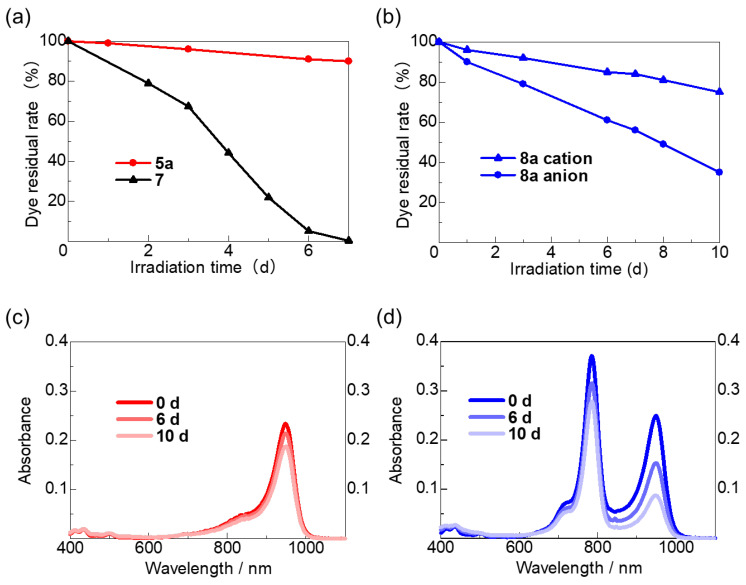
Photostabilities of the prepared anionic HMC dye **5a** and cyanine–cyanine mixed dye **8a** in CH_2_Cl_2_ (1 × 10^−6^ M) under white LED irradiation (8.5 W) in an incubator at 25 °C. Changes in the residual rates of (**a**) **5a** and cationic HMC dye **7**, and (**b**) **8a**, respectively. Changes in the absorption spectra of (**c**) **5a** and (**d**) **8a** over time.

The photostabilities of the anionic HMC dye **5a** and cyanine–cyanine mixed dye **8a** were evaluated by irradiating them with a white LED light (8.5 W, emitting blue LED + yellow phosphor, peak wavelength: 440 nm) in a CH_2_Cl_2_ solution (1.0 × 10^−6^ M) at 25 °C in a constant temperature chamber. The residual rates of dyes **5a** and **8a**, calculated from the change in absorbance at λ_max_ in the UV–vis–NIR spectra, are illustrated in Figure 3a–d, respectively, and are compared with those of previous HMC dyes (Table 2).

The residual rate of anionic HMC dye **5a** after 10 days of light irradiation was 80%. This dye showed the best photostability compared to the anionic and cationic HMC dyes we synthesized previously. The observed results can be ascribed to the enhanced electron-withdrawing properties of the trifluoromethyl groups. These groups significantly suppress the electrophilic addition of singlet oxygen to the methine chain [37]. 

Furthermore, when photostability tests were carried out on the cyanine–cyanine mixed dye **8a** under identical conditions, it was observed that the absorption peaks originating from the anionic HMC structure diminished more rapidly compared to those from the cationic HMC structure. This is because the methine chain of the anionic HMC skeleton, which is more electron-rich than the cationic skeleton, is more readily affected by the electrophilic addition to singlet oxygen, which matches the reported photodegradation mechanism. Interestingly, the photostability of the anionic dye in the cyanine–cyanine mixed dye **8a** was much lower than that of anionic HMC **5a** alone. In contrast, the photostability of the cationic dye in cyanine–cyanine mixed dye **8a** was significantly improved compared to that of cationic HMC **7** alone.

## 3. Experimental Section

### 3.1. Measurements

The ^1^H NMR spectra of the compounds were obtained at 392 or 400 MHz in CDCl_3_, hexadeuteroacetone ((CD_3_)_2_CO), or hexadeuterodimethyl sulfoxide ((CD_3_)_2_SO) solutions using the residual solvent as the internal standard and a JEOL ECS-400 or ECX-400P Fourier transform NMR (FT-NMR) spectrometer. The ^13^C NMR spectra of the compounds were obtained at 99 or 101 MHz in CDCl_3_, (CD_3_)_2_CO, or (CD_3_)_2_SO solutions, using the residual solvent as the internal standard, and a JEOL ECS-400 or ECX-400P FT-NMR spectrometer. The ^19^F NMR spectra of the compounds were obtained at 369 or 376 MHz in CDCl_3_ or (CD_3_)_2_CO solutions, respectively, using CFCl_3_ as the external standard and a JEOL ECS-400 or ECX-400P FT-NMR spectrometer. The data were reported as follows: (s = singlet, t = triplet, q = quartet, m = multiplet, br s = broad singlet, coupling constant(s), and integration). The melting points of the compounds were obtained using a Yanagimoto MP-S3 micro melting point apparatus and are uncorrected. The compounds’ infrared (IR) spectra were recorded on a Shimadzu IR Affinity-1 instrument. Electrospray ionization-mass spectroscopy (ESI–MS) and HRMS measurements were performed using a Waters Xevo quadrupole time-of-flight (QTOF) mass spectrometer. The UV–vis–NIR absorption spectra of the dyes in solution were recorded using a Hitachi U-4100 instrument. The CV profiles were obtained using an HSV-110 automatic polarization system. TG–DTA experiments were performed using an SII EXSTAR 6000 thermogravimetry differential thermal analysis (TG/DTA) 6300 apparatus under a nitrogen atmosphere after heating to 80 °C under vacuum for 18 h; the measured values were uncorrected.

### 3.2. Materials

Diacetyl, 2-hydroxy-2-(trifluoromethyl)propionitrile, iodomethane, malononitrile, tetrabutylammonium iodide, cyclohexanone, and 2,3,3-trimethyl-3*H*-indole were purchased from TCI Fine Chemicals. Bromopentafluorobenzene, *N*,*N*-dimethylformamide (DMF), phosphoryl chloride, super-dehydrated diethyl ether, and acetic anhydride were purchased from Wako Pure Chemicals. Hydrogen chloride (ca. 12 mol/L in water) and sodium acetate (AcONa) were purchased from NACALAI TESQUE, Inc., Shiga, Japan. Acetone was purchased from KANTO CHEMICAL CO., Japan. Isopropylmagnesium chloride lithium chloride complex solution, methylmagnesium bromide solution (ca. 3.0 mol/L in diethyl ether (Et_2_O)), and lithium ethoxide were purchased from Sigma-Aldrich Co. LLC, Tokyo, Japan.

Pure products were isolated via column chromatography using silica gel 60 (spherical, 270–325 mesh, Kanto Chemical Co., Inc.,Tokyo, Japan) or Wakogel^®^ C-200 (100–200 mesh, FUJIFILM Wako Pure Chemical Corporation, Osaka, Japan). Analytical thin-layer chromatography (TLC) was performed using Merck pre-coated (0.25 mm) silica gel 60 F_254_ plates. All chemicals were reagent-grade and purified before use if needed.

### 3.3. Synthesis of 4,4,4-Trifluoro-3-hydroxy-3-methylbutan-2-one (***1a***) [41]

2-Hydroxy-2-(trifluoromethyl)propionitrile (1.467 g, 10.125 mmol) was dissolved in Et_2_O super-dehydrated (14 mL) under an argon atmosphere. After that, 8.42 mL of methylmagnesium bromide (3 M in Et_2_O), which was cooled to −20 °C, was added dropwise to the solution. After the reaction mixture was warmed to 0 °C, it was added to 10% hydrochloric acid (60 mL) and stirred at 25 °C for 1 h. Subsequently, the reaction mixture was neutralized by adding saturated Na_2_CO_3_ *aq* (65 mL), and the blend was extracted with Et_2_O (30 mL × 3). Next, the combined organic layers were dried over anhydrous sodium sulfate (Na_2_SO_4_). The organic solvent was concentrated to obtain 4,4,4-trifluoro-3-hydroxy-3-methylbutan-2-one (**1a**) (1.368 g), used in subsequent reactions without further purification.

### 3.4. Synthesis of 2-(2-Cyano-3,4-dimethyl-4-(trifluoromethyl)cyclopent-2-en-1-ylidene)malononitrile (***2a***) [42]

First, a mixture of **1a** (0.601 g, 3.847 mmol) and malononitrile (0.510 g, 7.724 mmol) was dissolved in an anhydrous THF (4 mL) under an argon atmosphere. Subsequently, 0.269 mL of lithium ethoxide (1.0 M in EtOH, 0.269 mmol) was added dropwise to the solution. The reaction mixture was stirred under reflux for 8 h and then concentrated using a rotary evaporator. The residue was extracted with CH_2_Cl_2_ (30 mL × 3), washed with brine, and the combined organic layers were dried over anhydrous Na_2_SO_4_. After the organic solvent was concentrated, the crude product was purified via column chromatography on silica gel using CH_2_Cl_2_ as the solvent, followed by washing with methanol to produce 2-(2-cyano-3,4-dimethyl-4-(trifluoromethyl)cyclopent-2-en-1-ylidene)malononitrile (**2a**) with a yield of 25% (0.239 g).
White solid; Yield 25%; m.p. = 160.1–161.0 °C; *Rf* 0.49 (CH_2_Cl_2_); IR (KBr) 2233 (C≡N) cm^−1^; HRMS (ESI) found: *m*/*z* 254.0555. Calc. for C_16_H_12_N_3_O: 254.0541; ^1^H NMR (CDCl_3_) δ 1.84 (s, 3H, –C*H_3_*), 2.46 (s, 3H, –C*H_3_*); ^13^C NMR (CDCl_3_) δ 14.7 (s), 17.6 (s), 62.6 (s), 96.2 (q, *J* = 32.89 Hz), 107.9 (s), 109.1 (s), 109.5 (s), 109.7 (s), 121.6 (q, J = 285.94 Hz), 172.0 (s), 173.8 (s); ^19^F NMR (CDCl_3_) δ −77.23 (s, 3F) (see S2–S4 in the Supplementary Materials).

### 3.5. Synthesis of (E)-2-Chloro-3-(hydroxymethylene)cyclohex-1-ene-1-carbaldehyde (***3***) [44,48]

Phosphoryl chloride (1.90 mL, 20.119 mmol) was added dropwise to DMF (4 mL) at 0 °C under an argon atmosphere, and the reaction mixture was stirred for 30 min. A DMF (1 mL) solution of cyclohexanone (0.514 g, 5.080 mmol) was slowly added to the mixture at 0 °C, followed by stirring for 30 min. After the reaction mixture was heated to 55 °C, it was stirred for 4 h. Next, 150 mL of water and crushed ice was added to the reaction mixture, and the blend was stored in a refrigerator overnight. The precipitate obtained was filtered and washed with water. The yield of the prepared solid, name (*E*)-2-chloro-3-(hydroxymethylene)cyclohex-1-ene-1-carbaldehyde (**3**), was 67% (0.595 g).
Yellow solid; Yield 67%; m.p. = 143.0–145.0 °C; IR (KBr) 1620 (C=O) cm^−1^; HRMS (ESI) found: *m*/*z* 173.0370. Calcd for C_8_H_9_ClO_2_: 173.0369; ^1^H NMR (DMSO-*d*_6_) δ 1.57 (quin, *J* = 6.17 Hz, 2H, –CH_2_C*H*_2_CH_2_–), 2.35 (s, 4H, –C*H*_2_CH_2_C*H*_2_–), 7.56 (br s, 1H, vinly *H*), 10.1 (br s, 1H, –C*H*O), 10.8 (br s, 1H, –O*H*); ^13^C NMR (DMSO-*d*_6_) δ 20.0 (s), 23.7 (s), 146.1 (s) (see S5 and S6 in the Supplementary Materials).

### 3.6. Synthesis of Sodium ((Z)-4-((E)-2-(2-Chloro-3-((E)-2-(4-cyano-5-(dicyanomethylene)-2-methyl-2-(trifluoromethyl)-2,5-dihydrofuran-3-yl)vinyl)cyclohex-2-en-1-ylidene)ethylidene)-3-cyano-5-methyl-5-(trifluoromethyl)-4,5-dihydrofuran-2-yl)dicyanomethanide (***4a***)

A mixture of **2a** (0.205 g, 0.810 mmol), **3** (0.069 g, 0.401 mmol), and sodium acetate (0.075 g, 0.890 mmol) in acetic anhydride (8 mL) was stirred at 25 ℃ overnight under an argon atmosphere. The reaction mixture was added to hexane (200 mL) and Et_2_O (15 mL), and the precipitate was filtered. The dark green solid crude product, namely sodium ((*Z*)-4-((*E*)-2-(2-chloro-3-((*E*)-2-(4-cyano-5-(dicyanomethylene)-2-methyl-2-(trifluoromethyl)-2,5-dihydrofuran-3-yl)vinyl)cyclohex-2-en-1-ylidene)ethylidene)-3-cyano-5-methyl-5-(trifluoromethyl)-4,5-dihydrofuran-2-yl)dicyanomethanide (**4a**) (0.273 g), was used for subsequent reactions without further purification.

### 3.7. Synthesis of Tetrabutylammonium ((Z)-4-((E)-2-(2-Chloro-3-((E)-2-(4-cyano-5-(dicyanomethylene)-2-methyl-2-(trifluoromethyl)-2,5-dihydrofuran-3-yl)vinyl)cyclohex-2-en-1-ylidene)ethylidene)-3-cyano-5-methyl-5-(trifluoromethyl)-4,5-dihydrofuran-2-yl)dicyanomethanide (***5a***)

A mixture of **4a** (0.273 g, 0.411 mmol) and tetrabutylammonium iodide (0.039 g, 0.452 mmol) in acetone (7 mL) was stirred at 25 °C for 2 h under an argon atmosphere. After the solvent was removed under low pressure, the residue was purified via column chromatography on silica gel using a CH_2_Cl_2_–methanol (100:1 (*v*/*v*)) solvent to obtain tetrabutylammonium ((*Z*)-4-((*E*)-2-(2-chloro-3-((*E*)-2-(4-cyano-5-(dicyanomethylene)-2-methyl-2-(trifluoromethyl)-2,5-dihydrofuran-3-yl)vinyl)cyclohex-2-en-1-ylidene)ethylidene)-3-cyano-5-methyl-5-(trifluoromethyl)-4,5-dihydrofuran-2-yl)dicyanomethanide (**5a**) with a yield of 13% (0.047 g).
Dark green solid; Yield 13%; *T*_dt_ 238.3 °C; *R_f_* 0.46 (CH_2_Cl_2_/methanol = 20/1); IR (KBr) 2214 (C≡N) cm^−1^; HRMS (ESI) found: *m*/*z* 641.0933. Calc. for C_30_H_16_ClF_6_N_6_O_2_: Bu_4_N, 641.0927; ^1^H NMR (Acetone-*d*_6_) δ 0.98 (td, *J* = 7.31, 1.35 Hz, 12H, –CH_2_CH_2_CH_2_C*H*_3_ × 4), 1.44 (sex, *J* = 7.31 Hz, 8H, –CH_2_CH_2_C*H*_2_CH_3_ × 4), 1.77–1.80 (m, 2H, –CH_2_C*H*_2_CH_2_–), 1.80–1.86 (m, 8H, –CH_2_C*H*_2_CH_2_CH_3_ × 4), 1.93 (s, 6H, –C*H*_3_ × 2), 2.59–2.73 (m, 4H, –C*H*_2_CH_2_C*H*_2_–), 3.41–3.45 (m, 8H, –C*H*_2_CH_2_CH_2_CH_3_ × 4), 6.24 (d, *J* = 13.91 Hz, 2H, vinyl *H*), 8.58 (d, *J* = 13.91 Hz, 2H, vinyl *H*); ^13^C NMR (Acetone-*d*_6_) δ 13.9 (s), 19.7 (s), 20.4 (s), 21.6 (s), 24.4 (s), 26.9 (s), 49.6 (s), 59.4 (s), 85.7 (s), 92.9 (q, *J* = 31.63 Hz), 109.4 (s), 114.0 (s), 114.6 (s), 124.0 (q, *J* = 282.50 Hz), 131.4(s), 137.9 (s), 141.7 (s), 149.4 (s), 156.6 (s), 177.2 (s); ^19^F NMR (Acetone-*d*_6_) δ −80.29 (s, 6F) (see S7–S9 and S18 in the Supplementary Materials).

### 3.8. Synthesis of 1,2,3,3-Tetramethyl-3H-indol-1-ium Iodide (***6***) [47]

To an acetonitrile solution (5 mL) of 2,3,3-trimethyl-3*H*-indole (0.7883 g, 4.950 mmol), iodomethane was added (1.417 g, 9.980 mmol), and the mixture was stirred at 40 °C for 1 day. After the reaction mixture was poured into diethyl ether (75 mL), the precipitate was filtered to 1,2,3,3-tetramethyl-3H-indol-1-ium iodide (3) (1.180 g, 82%).
Pale pink solid; Yield 82%; m.p. = 248.0–252.0 °C; IR (KBr) 1609 (C=N) cm^−1^; HRMS (ESI) found: *m*/*z* 174.1283 Calc. for C_12_H_16_IN: [M-I]^+^, 174.1254; ^1^H NMR (DMSO-*d_6_*) δ 1.54 (s, 6H, –CH(C*H_3_*)(C*H_3_*)), 2.79–2.80 (m, 3H, C*H_3_*), 3.99 (s, 3H, -N*CH_3_*_)_, 7.58–7.65 (m, 2H, aryl H × 2), 7.81–7.88 (m, 1H, aryl H), 7.89–7.95 (m, 1H, aryl H); ^13^C NMR (DMSO-*d_6_*) δ 14.5 (s), 21.7 (s), 34.9 (s), 53.9 (s), 115.1 (s), 123.3 (s), 128.8 (s), 129.2 (s), 141.6 (s), 142.1 (s), 195.9 (s) (see S10 and S11 in the Supplementary Materials).

### 3.9. Synthesis of 2-((E)-2-((E)-2-Chloro-3-(2-((E)-1,3,3-trimethylindolin-2-ylidene)ethylidene)cyclohex-1-en-1-yl)vinyl)-1,3,3-trimethyl-3H-indol-1-ium Iodide (***7***) [46]

(*E*)-2-chloro-3-(hydroxymethylene)cyclohex-1-ene-1-carbaldehyde (**3**) (0.085 g, 0.492 mmol) was added to a *N,N*-dimethylformamide solution (3 mL) of 1,2,3,3-tetramethyl-3*H*-indol-1-ium iodide (**6**) (0.3036 g, 1.001 mmol), and the mixture was stirred at 120 °C for 5 h. The reaction mixture was poured into ice water (100 mL), stirred for 30 min, and thereafter refrigerated for 30 min. The resulting precipitate was collected using suction filtration. The residue was purified using silica gel chromatography (dichloromethane/methanol = 25/1) to yield 2-((*E*)-2-((*E*)-2-chloro-3-(2-((*E*)-1,3,3-trimethylindolin-2-ylidene)ethylidene)cyclohex-1-en-1-yl)vinyl)-1,3,3-trimethyl-3*H*-indol-1-ium iodide (**7**) (0.229 g, 76%).
Yellow-green solid; Yield 76%; *T*_dt_ 244.0 °C; *R_f_* 0.25 (CH_2_Cl_2_/methanol = 25/1); IR (KBr) 1550 (C=N) cm^−1^; HRMS (ESI) found: *m*/*z* 483.2561 Calc. for C_32_H_36_N_2_Cl: [M − I^−^]^+^, 483.2567; ^1^H NMR (CDCl_3_) δ 1.77 (s, 12H, –C(C*H_3_*)_2_ × 2), 1.96–1.99 (m, 2H, –CH_2_C*H*_2_CH_2_–), 2.75–2.81 (m, 4H, –C*H*_2_CH_2_C*H_2_*–), 3.76 (s, 6H, NC*H_3_* × 2), 6.25 (d, *J* = 13.91 Hz, 2H, vinyl H×2), 7.17–7.24 (m, 4H, Aryl H), 7.34–7.39 (m, 4H, Aryl H), 8.34 (d, *J* = 13.91 Hz, 2H, vinyl H×2); ^13^C NMR (CDCl_3_) δ 20.8 (s), 27.0 (s), 28.2 (s), 32.8 (s), 49.3 (s), 102.0 (s), 110.9 (s), 122.2 (s), 125.4 (s), 128.1 (s), 129.0 (s), 141.1 (s), 143.0 (s), 144.4 (s), 150.6 (s), 173.0 (s) (see S12, S13 and S18 in the Supplementary Materials).

### 3.10. Synthesis of 2-((E)-2-((E)-2-Chloro-3-(2-((E)-1,3,3-trimethylindolin-2-ylidene)ethylidene)cyclohex-1-en-1-yl)vinyl)-1,3,3-trimethyl-3H-indol-1-ium ((Z)-4-((E)-2-(2-chloro-3-((E)-2-(4-cyano-5-(dicyanomethylene)-2-methyl-2-(trifluoromethyl)-2,5-dihydrofuran-3-yl)vinyl)cyclohex-2-en-1-ylidene)ethylidene)-3-cyano-5-methyl-5-(trifluoromethyl)-4,5-dihydrofuran-2-yl)dicyanomethanide (***8a***)

A mixture of **7** (0.008 g, 0.013 mmol) and **5a** (0.009 g, 0.0010 mmol) in acetone (2 mL) was stirred at 25 ℃ overnight under an argon atmosphere. After the solvent was removed under low pressure, the residue was purified via column chromatography on silica gel using a CH_2_Cl_2_–methanol (200:1 (*v*/*v*)) solvent to obtain 2-((*E*)-2-((*E*)-2-chloro-3-(2-((*E*)-1,3,3-trimethylindolin-2-ylidene)ethylidene)cyclohex-1-en-1-yl)vinyl)-1,3,3-trimethyl-3*H*-indol-1-ium ((*Z*)-4-((*E*)-2-(2-chloro-3-((*E*)-2-(4-cyano-5-(dicyanomethylene)-2-methyl-2-(trifluoromethyl)-2,5-dihydrofuran-3-yl)vinyl)cyclohex-2-en-1-ylidene)ethylidene)-3-cyano-5-methyl-5-(trifluoromethyl)-4,5-dihydrofuran-2-yl)dicyanomethanide (**8a**) with a yield of 92% (0.011 g).
Red glossy solid; Yield 92%; *T*_dt_ 201.3 °C; *R_f_* 0.55 (CH_2_Cl_2_/methanol = 10/1); IR (KBr) 1550 (C=N), 2214 (C≡N) cm^−1^; HRMS (ESI) found: *m*/*z* 483.2566. Calc. for C_32_H_36_ClN_2_: [M]^+^, 483.2567, *m*/*z* 641.0931. Calc. for C_30_H_16_ClN_6_O_2_F_6_: [M]^−^, 641.0927; ^1^H NMR (Acetone-*d*_6_) δ 1.78 (s, 12H, –C*H*_3_ × 4), 1.83–1.88 (m, 2H, –CH_2_C*H*_2_CH_2_–), 1.93 (m, 6H, –C*H*_3_ × 2), 1.94–1.99 (m, 2H, –CH_2_C*H*_2_CH_2_–), 2.55–2.71 (m, 4H, –C*H*_2_CH_2_C*H*_2_–), 2.77 (t, *J* = 5.17 Hz, 4H, –C*H*_2_CH_2_C*H*_2_–), 3.80 (s, 3H, -NC*H*_3_ × 2), 6.24 (d, *J* = 13.91 Hz, 2H, vinyl *H*), 6.41 (d, *J* = 14.39 Hz, 2H, vinyl *H*), 7.32 (t, *J* = 6.73 Hz, 2H, aryl *H*), 7.41–7.49 (m, 4H, aryl *H*), 7.62 (d, *J* = 7.18 Hz, 2H, aryl *H*), 8.44 (d, *J* = 13.91 Hz, 2H, vinyl *H*), 8.55 (d, *J* = 14.39 Hz, 2H, vinyl *H*); ^13^C NMR (Acetone-*d*_6_) δ 19.6 (s), 21.6 (s), 26.9 (s), 27.0 (s), 28.1 (s), 32.0 (s), 49.6 (s), 50.1 (s), 85.8 (s), 92.8 (q, *J* = 31.32 Hz), 102.5 (s), 109.3 (s), 112.0 (s), 113.9 (s), 114.5 (s), 114.6 (s), 123.2 (s), 123.9 (q, *J* = 285.67 Hz), 126.2 (s), 127.4 (s), 130.0 (s), 131.3 (s), 141.6 (s), 142.1 (s), 144.1 (s), 144.7 (s), 149.4 (s), 150.1 (s), 156.7 (s), 174.3 (s), 177.1 (s); ^19^F NMR (Acetone-*d*_6_) δ −80.25 (s, 6F) (see S14–S16 and S18 in the Supplementary Materials).

### 3.11. Electrochemical Measurements of the Dyes

Electrochemical measurements of the dyes were performed in MeCN solutions (1.0 × 10^−3^ M) containing Bu_4_NClO_4_ (0.1 M). The *E*_ox_ values were measured using three small electrodes. A silver quasi-reference electrode, a platinum wire, and a carbon electrode were used as the reference, counter, and working electrodes, respectively. All the electrode potentials were calibrated concerning the Fc/ferrocenium redox couple. Electrochemical measurements were performed at a scan rate of 200 mV s^−1^. The *E*_ox_ value of Fc vs. SCE was 0.380 V [48]. The *E*_ox_ values vs. SCE were determined using the observed *E*_ox_ (V vs. Ag) values of the dyes in MeCN solutions as follows:

*E*_ox_ (V vs. SCE) = *E* (V vs. Ag, observed value) + 0.380 − (measured *E*_ox_ value of Fc for Ag in the MeCN solution). (1)

The energy of the HOMO (eV) was obtained using the *E*_ox_ (V vs. SCE) values as follows [48]:

HOMO (eV) = −(*E*_ox_ (V vs. SCE) + 4.4) (2)

The band gap (*E*_0-0_) and energy of the LUMO (eV) were calculated using the *λ*_onset_^abs^ value as follows:

*E*_0-0_ (eV) = 1240/*λ*_onset_^abs^ (nm) (3)

LUMO (eV) = HOMO (eV) − *E*_0-0_ (eV) (4)

### 3.12. Methods for Evaluating Photostability

CH_2_Cl_2_ solutions of the dyes were maintained in an incubator at 25 °C and irradiated with white LED light (8.5 W).

## 4. Conclusions

We synthesized a novel anionic HMC dye **5a** with trifluoromethyl groups in the dye skeleton and compared its properties with those of our previously synthesized anionic HMC dyes. The new anionic HMC dye **5a** showed a more red-shifted absorption wavelength and improved photostability than our previously synthesized anionic HMC dyes. These properties are attributed to the electron-withdrawing characteristics of the trifluoromethyl groups of **5a**, which are more potent than the substituents of the previously synthesized dyes. 

We proceeded to synthesize a cyanine–cyanine mixed dye, **8a**, composed of an anionic HMC skeleton bearing trifluoromethyl groups and a cationic HMC skeleton. Subsequent investigations into its absorption properties and photostability were conducted. Our findings revealed that the unique properties of both the anionic and cationic HMC skeletons were distinctly represented in this compound. In the cyanine–cyanine mixed dye **8a**, the photostability of the cationic HMC skeleton was enhanced by the photodegradation of the anionic HMC skeleton. The enhanced properties of compounds **5a** and **8a** indicated their potential suitability for photovoltaic devices, thus presenting a significant advantage. 

We are currently investigating organic solar cells that utilize only near-infrared light using HMC dyes with CF_3_ groups.

## Figures and Tables

**Table 1:** UV–vis–NIR absorption spectra of anionic HMC dyes **5a**–**d**, cationic HMC dye **7**, cyanine–cyanine mixed dye **8a** in CH_2_Cl_2_, and electrochemical properties of each anionic HMC dye **5a**–**d** in acetonitrile.

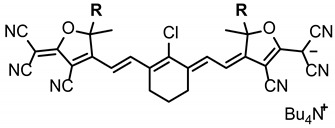									
Dye	R	λ_max_ (nm) ^a^	ε (M^−1^ cm^−1^) ^a^	*E*_ox_ (V vs. SCE) ^b^	HOMO (eV)	λ_onset_^abs^ (nm) ^a^	HOMO–LUMO Gap (eV) ^c^	LUMO (eV) ^d^	Reference
**5a**	CF_3_	948	230,000	0.57	−4.97	1080	1.15	−3.82	This study
**8a**	―	785 949	370,000 250,000	―	―	―	―	―	
**5b**	C_6_F_5_	934	310,000	0.43	−4.83	1053	1.18	−3.65	[37]
**5c**	Ph	920	330,000	0.34	−4.74	1033	1.20	−3.54	
**5d**	Me	906	260,000	0.30	−4.70	1024	1.21	−3.49	
**7**	―	785	380,000	―	―	―	―	―	

^a^ Measured in CH_2_Cl_2_ (1 × 10^−6^). ^b^ Measured in acetonitrile containing the dye (1.0 × 10^−3^ M) and Bu_4_NClO_4_ (0.1 M) and recalculated into *E*_ox_ (V vs. SCE) [48] (see S17 in the Supplementary Materials). ^c^ HOMO–LUMO gap (eV) = 1240/λ_onset_^abs^ (nm). ^d^ LUMO (eV) = HOMO − (HOMO–LUMO gap).

**Table 2:** Residual rates of anionic HMC dyes **5a**–**d**, cationic HMC dye **7**, and cyanine–cyanine mixed dye **8a** in CH_2_Cl_2_ after 10 d.

Dye	R	Residual Rates (%) ^a^		Reference
		Anion	Cation	
**5a**	CF_3_	80	―	This study
**8a**	―	35	75	
**5b**	C_6_F_5_	79	―	[37]
**5c**	Ph	62	―	
**5d**	Me	24	―	
**7**	―	―	0	

^a^ Measured in CH_2_Cl_2_ (1 × 10^−6^ M).

## Data Availability

The data presented in this study are available on request from the corresponding author.

## Supplementary Materials

The following supporting information can be downloaded at: https://www.mdpi.com/article/10.3390/molecules28124650/s1, NMR, IR, HRMS, Cyclic voltammograms, and TG–DTA.

## Sample Availability

Samples of the compounds are not available from the authors.
