# Supplementary material for: Synthesis of Near-Infrared-Absorbing Anionic Heptamethine Cyanine Dyes with Trifluoromethyl Groups
Source: Molecules. 2023 Jun 8;28(12):4650. doi: 10.3390/molecules28124650 (PMC10303145; doi:10.3390/molecules28124650)
Supplement: Supplementary file 1 [file molecules-28-04650-s001.zip › molecules-2429266-supplementary.pdf]

## (Contents)

$^{1}H$, $^{13}C$, $^{19}F$ NMR, IR and HRMS for **2a** ............ S2–S4

$^{1}H$, $^{13}C$ NMR, IR and HRMS for **3** ............ S5, S6

$^{1}H$, $^{13}C$, $^{19}F$ NMR, IR and HRMS for **5a** ............ S7–S9

$^{1}H$, $^{13}C$ NMR, IR and HRMS for **6** ............ S10, S11

$^{1}H$, $^{13}C$ NMR, IR and HRMS for **7** ............ S12, S13

$^{1}H$, $^{13}C$, $^{19}F$ NMR, IR and HRMS for **8a** ............ S14–S16

Result of CV measurement for **5a** ............ S17

Results of TG–DTA experiments for **5a**, **7a** ............ S18

**2-(2-Cyano-3,4-dimethyl-4-(trifluoromethyl)cyclopent-2-en-1-ylidene)malononitrile (2a) [1]**

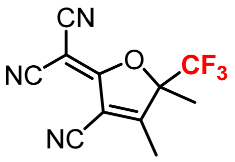


**2a**

**$^{1}$H NMR**

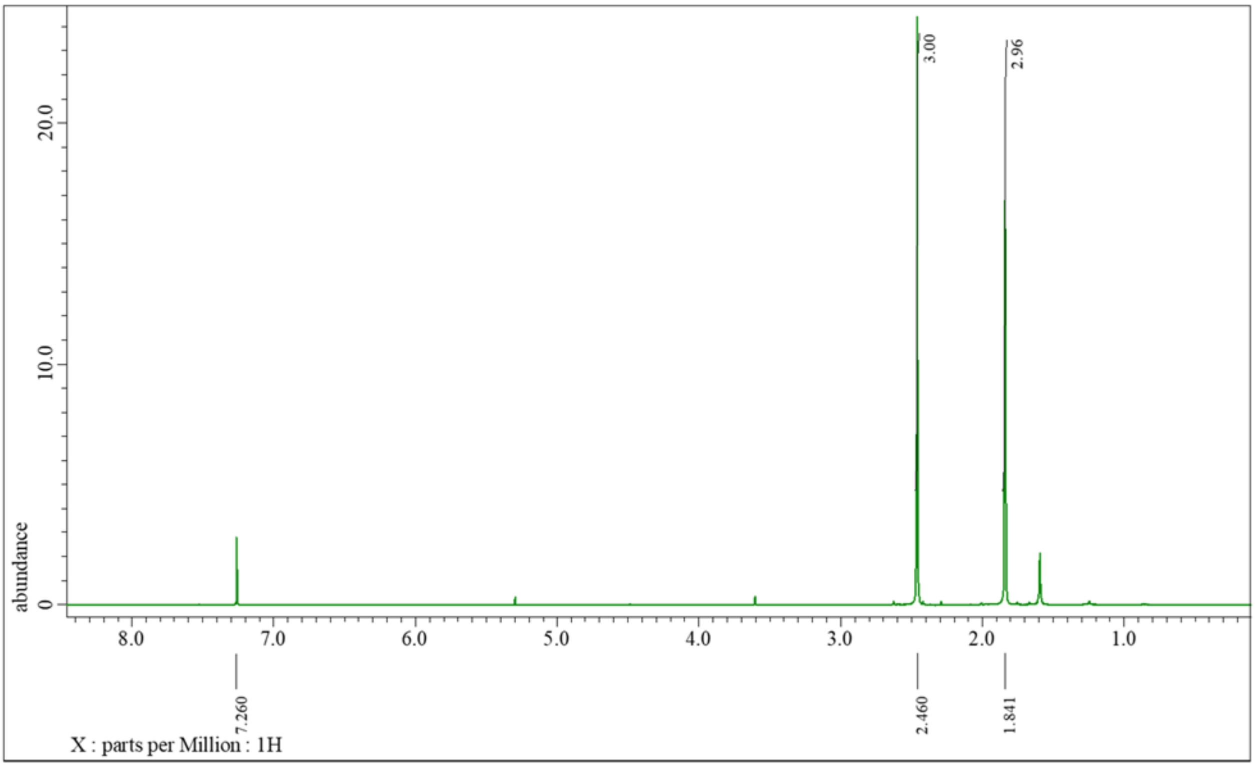


**$^{13}$C NMR**

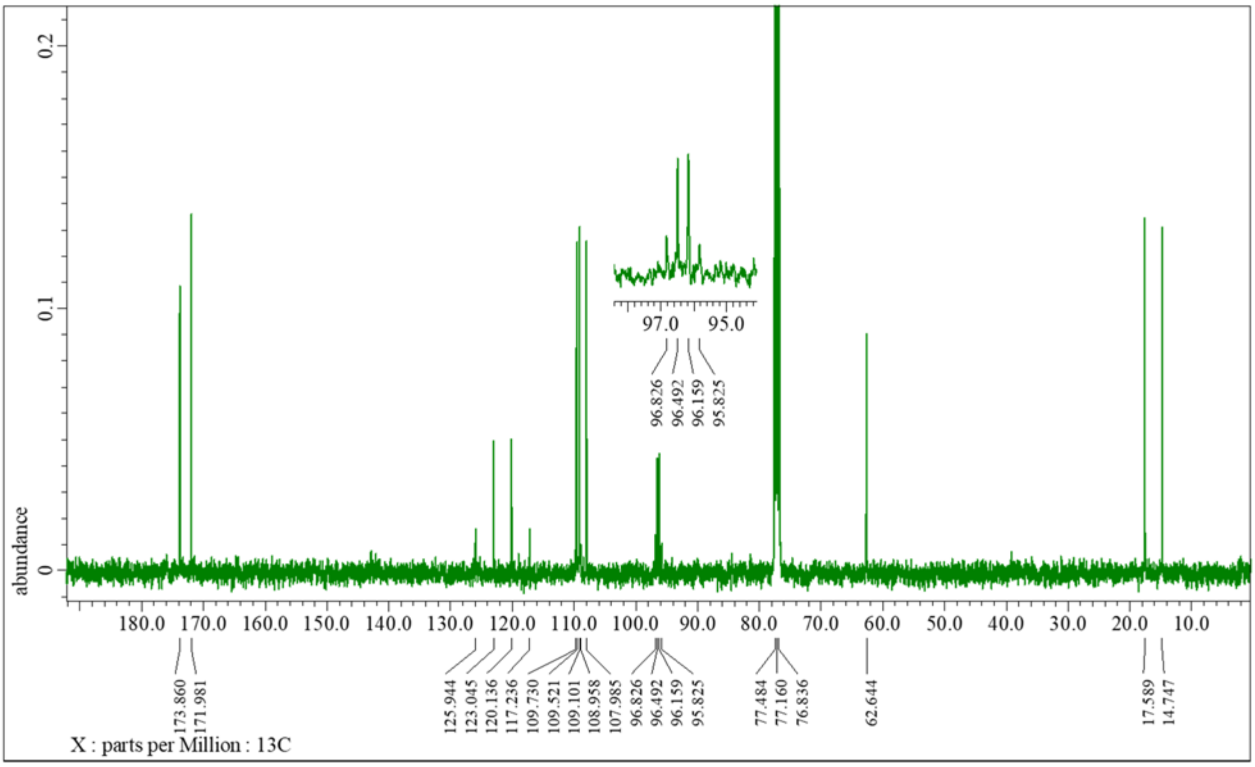

$^{19}$F NMR

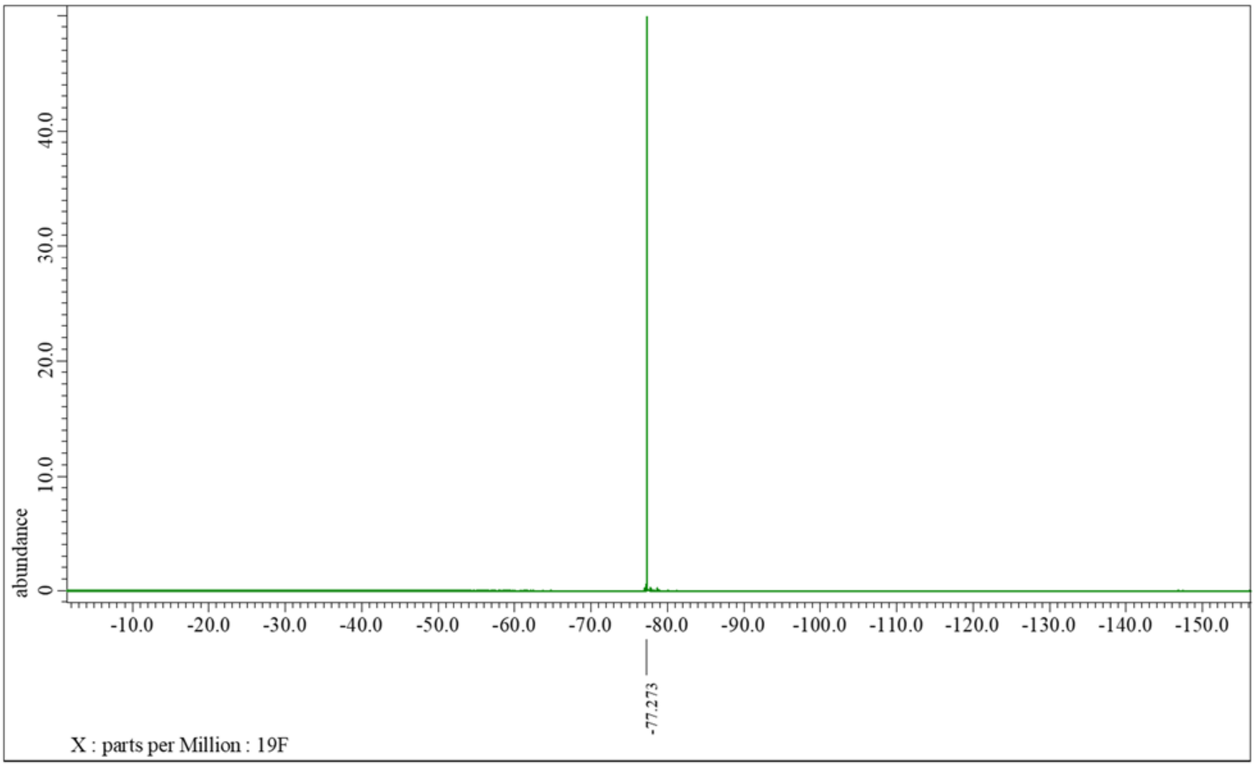

abundance
0
10.0
20.0
30.0
40.0
-10.0
-20.0
-30.0
-40.0
-50.0
-60.0
-70.0
-80.0
-90.0
-100.0
-110.0
-120.0
-130.0
-140.0
-150.0
-77.273
X : parts per Million : 19F


IR

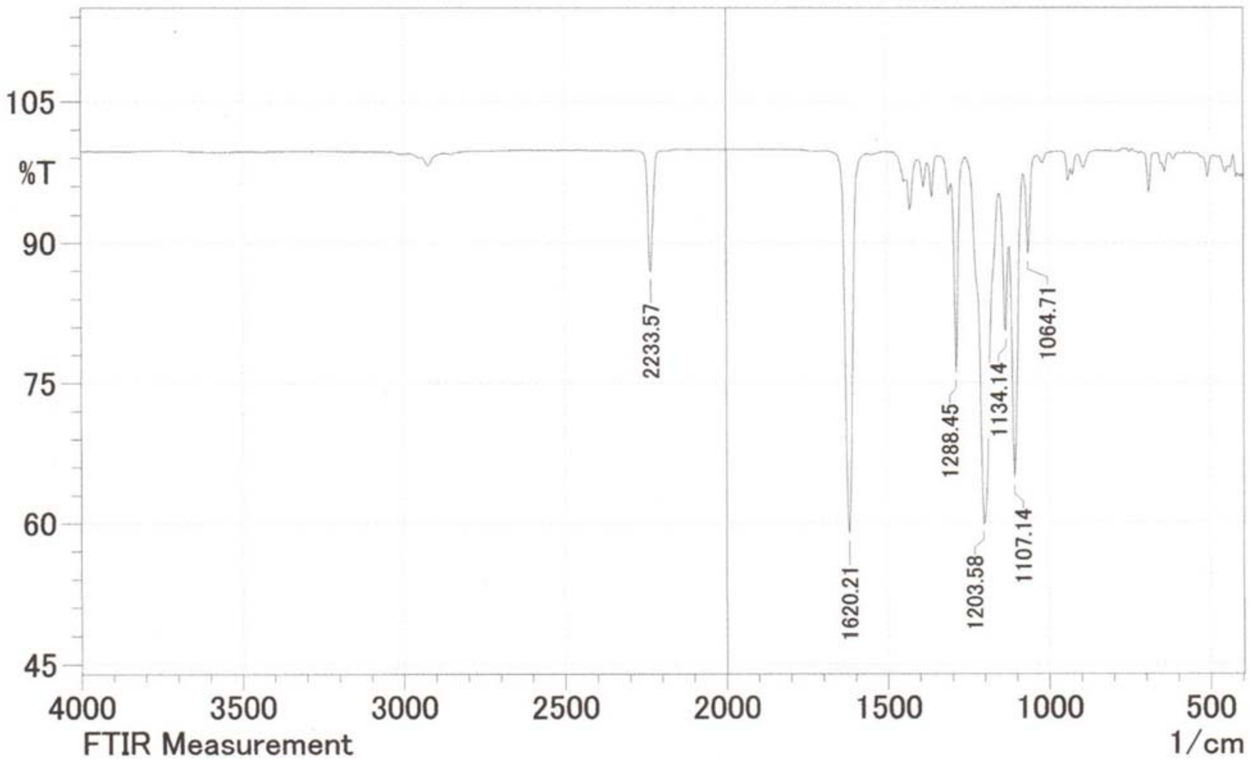

105
%T
90
75
60
45
2233.57
1620.21
1288.45
1203.58
1134.14
1107.14
1064.71
4000
3500
3000
2500
2000
1500
1000
500
FTIR Measurement
1/cm

**HRMS**

## Elemental Composition Report

Page 1

### Single Mass Analysis

Tolerance = 50.0 mDa / DBE: min = -1.5, max = 100.0
Element prediction: Off
Number of isotope peaks used for i-FIT = 3

Monoisotopic Mass, Even Electron Ions
4 formula(e) evaluated with 1 results within limits (up to 50 best isotopic matches for each mass)
Elements Used:
C: 11-11 H: 6-7 N: 3-3 O: 1-1 F: 3-3 Na: 0-1 Cl: 0-1

20220422_2 15 (0.524)
1: TOF MS ES+

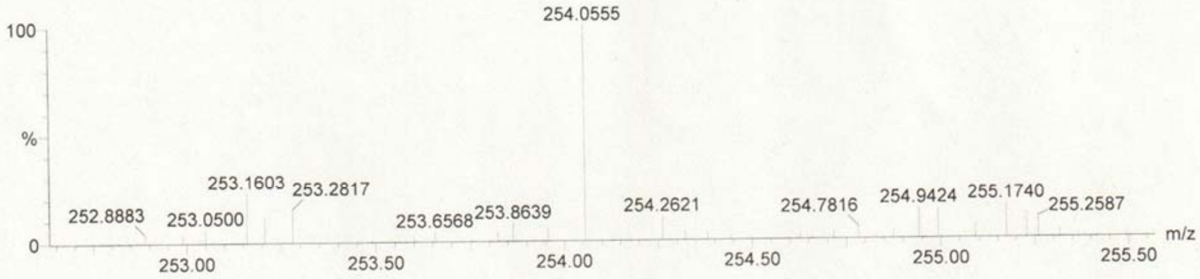


| | | | | | | | |
|---|---|---|---|---|---|---|---|
| Minimum: | | | | -1.5 | | | |
| Maximum: | | 50.0 | 80.0 | 100.0 | | | |
| Mass | Calc. Mass | mDa | PPM | DBE | i-FIT | i-FIT (Norm) | Formula |
| 254.0555 | 254.0541 | 1.4 | 5.5 | 8.5 | 67.1 | 0.0 | C11 H7 N3 O F3 |

**(*E*)-2-Chloro-3-(hydroxymethylene)cyclohex-1-ene-1-carbaldehyde (3) [2]**

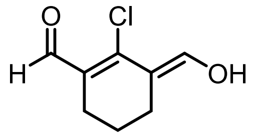


**3**

**$^{1}$H NMR**

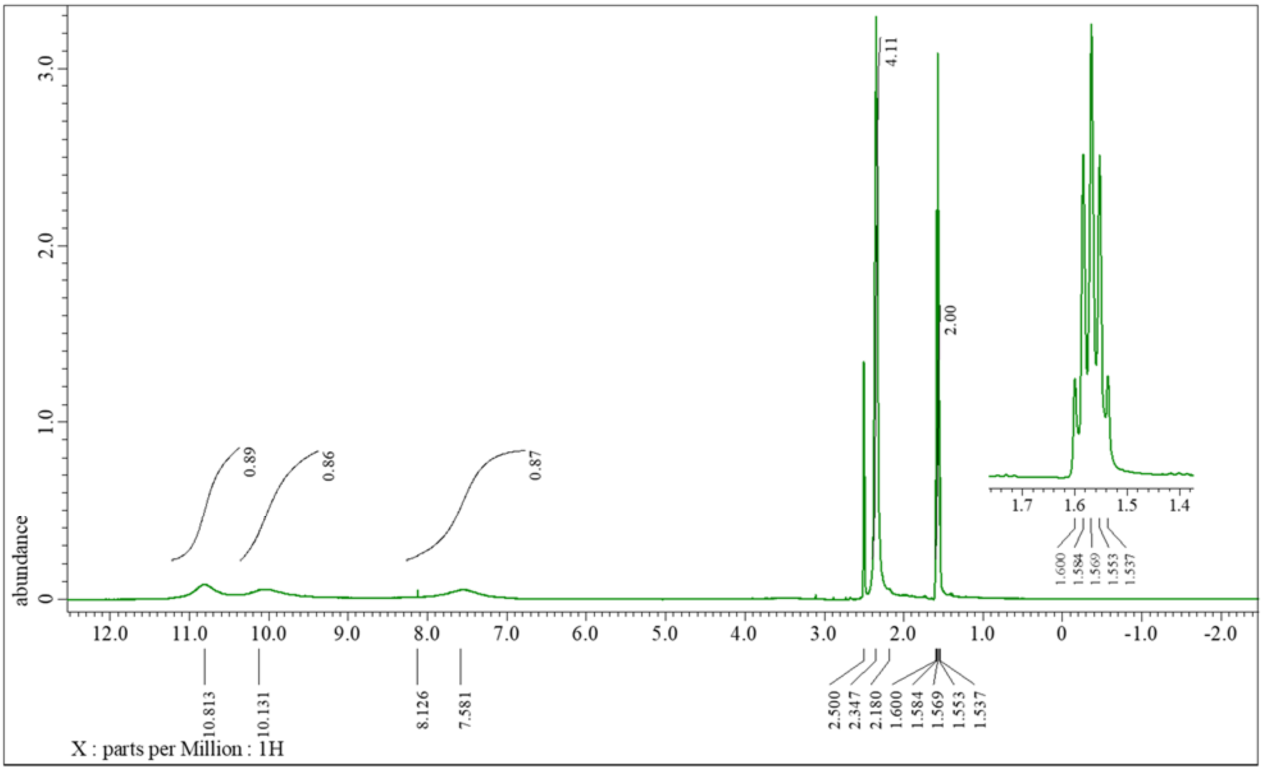


**$^{13}$C NMR**

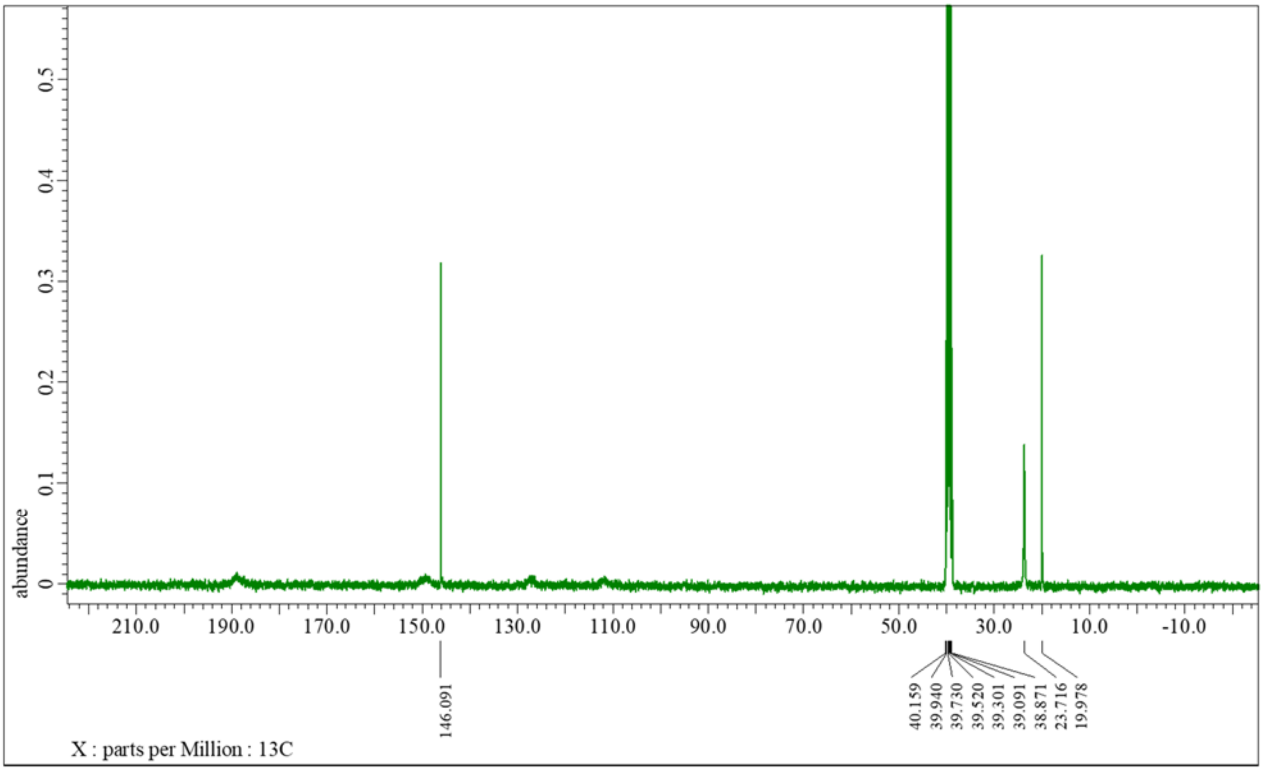

**IR**

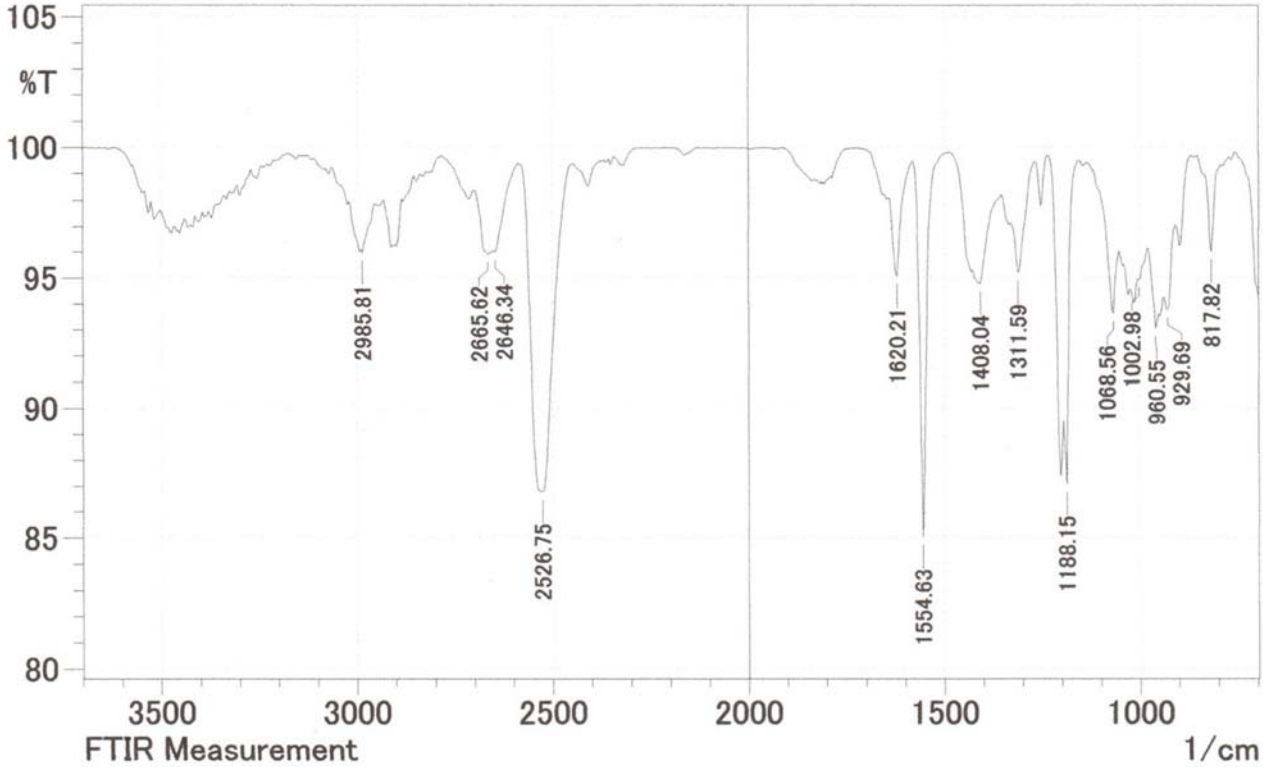


**HRMS**

**Elemental Composition Report** **Page 1**

**Single Mass Analysis**
Tolerance = 50.0 mDa / DBE: min = -1.5, max = 100.0
Element prediction: Off
Number of isotope peaks used for i-FIT = 3

Monoisotopic Mass, Even Electron Ions
2 formula(e) evaluated with 1 results within limits (up to 50 best isotopic matches for each mass)
Elements Used:
C: 8-8 H: 9-10 O: 2-2 Na: 0-1 Cl: 1-1
20230422_8 8 (0.284)
1: TOF MS ES+

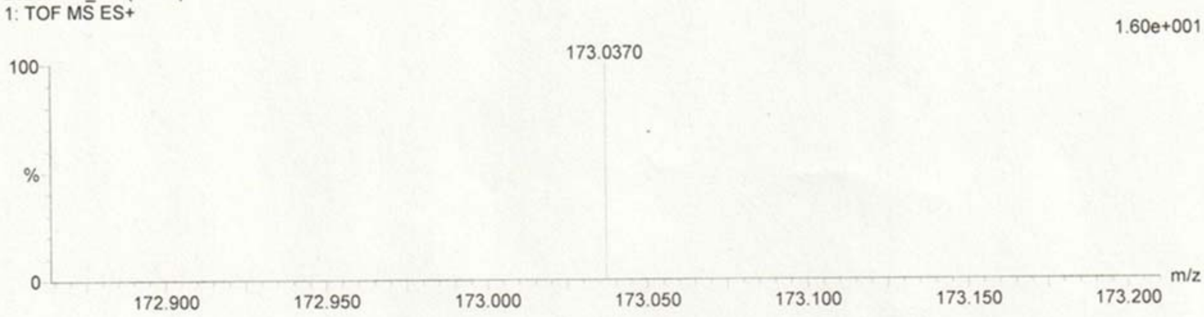


| | | | | | | | |
|---|---|---|---|---|---|---|---|
| Minimum: | | | | -1.5 | | | |
| Maximum: | | 50.0 | 80.0 | 100.0 | | | |
| Mass | Calc. Mass | mDa | PPM | DBE | i-FIT | i-FIT (Norm) | Formula |
| 173.0370 | 173.0369 | 0.1 | 0.6 | 3.5 | 11.6 | 0.0 | C8 H10 O2 Cl |

**Tetrabutylammonium ((*Z*)-4-((*E*)-2-(2-chloro-3-((*E*)-2-(4-cyano-5-(dicyanomethylene)-2-methyl-2-(trifluoromethyl)-2,5-dihydrofuran-3-yl)vinyl)cyclohex-2-en-1-ylidene)ethylidene)-3-cyano-5-methyl-5-(trifluoromethyl)-4,5-dihydrofuran-2-yl)dicyanomethanide (5a)**

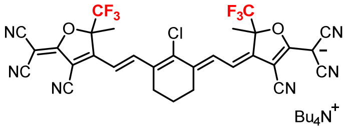


5a

$^{1}$H NMR

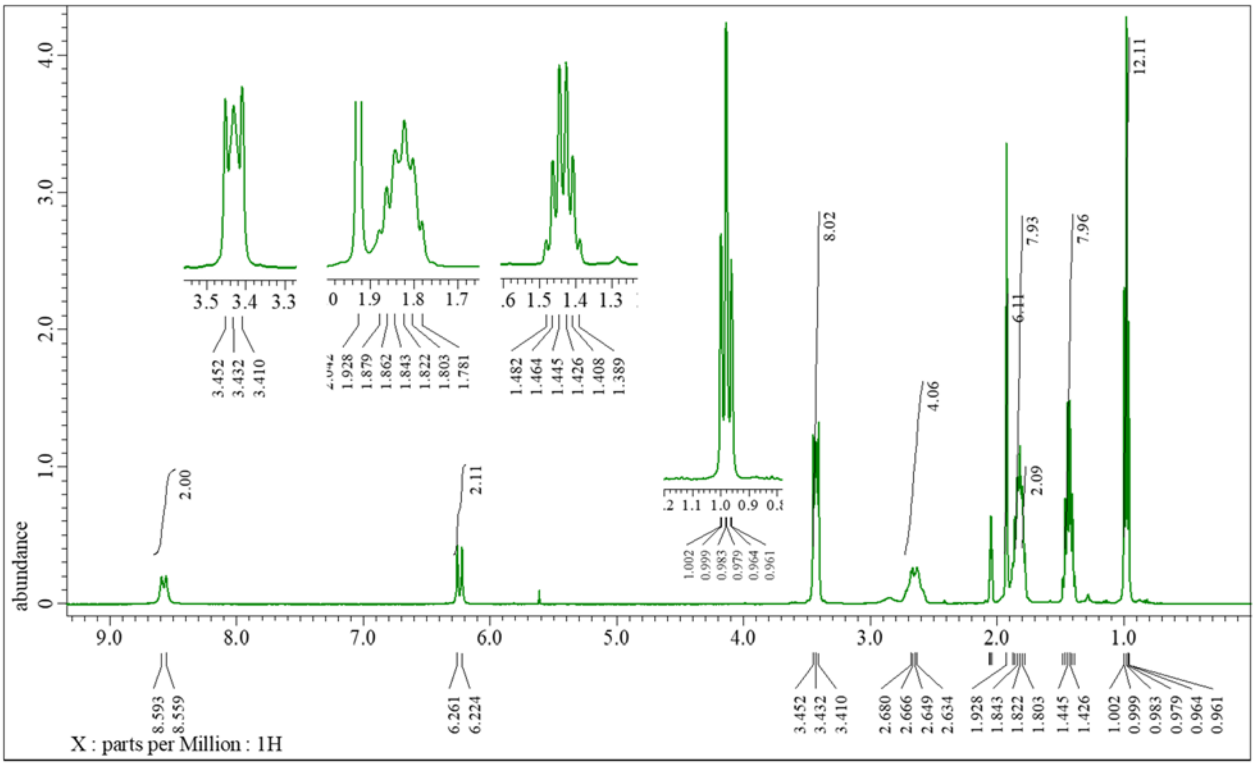


$^{13}$C NMR

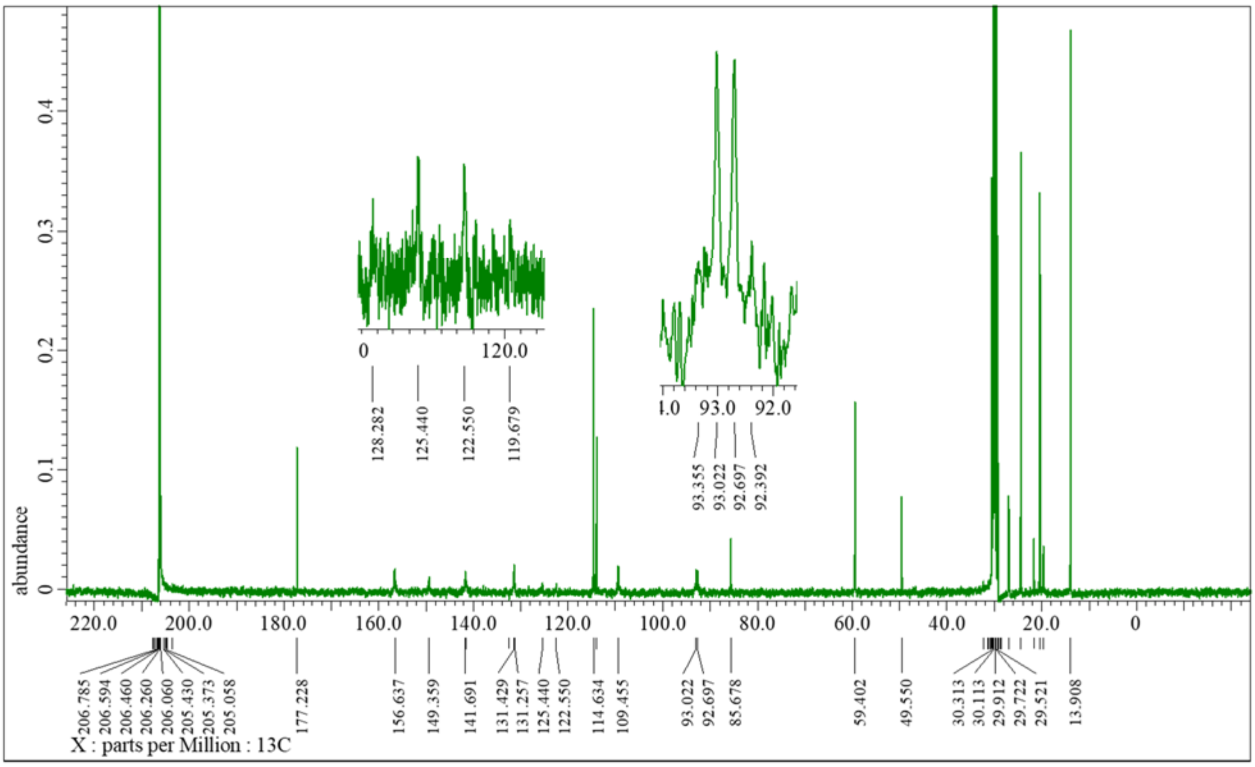

$^{19}F$ NMR

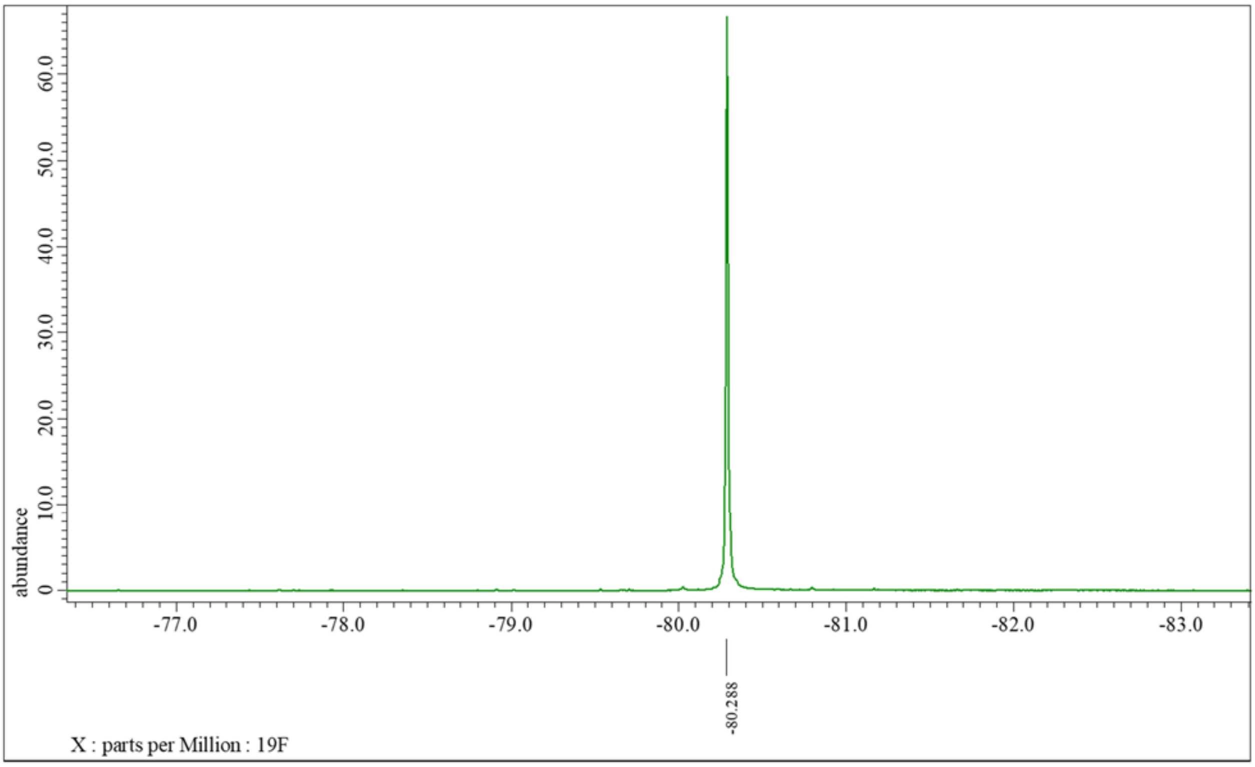

abundance
0
10.0
20.0
30.0
40.0
50.0
60.0
-77.0
-78.0
-79.0
-80.0
-81.0
-82.0
-83.0
-80.288
X : parts per Million : 19F


IR

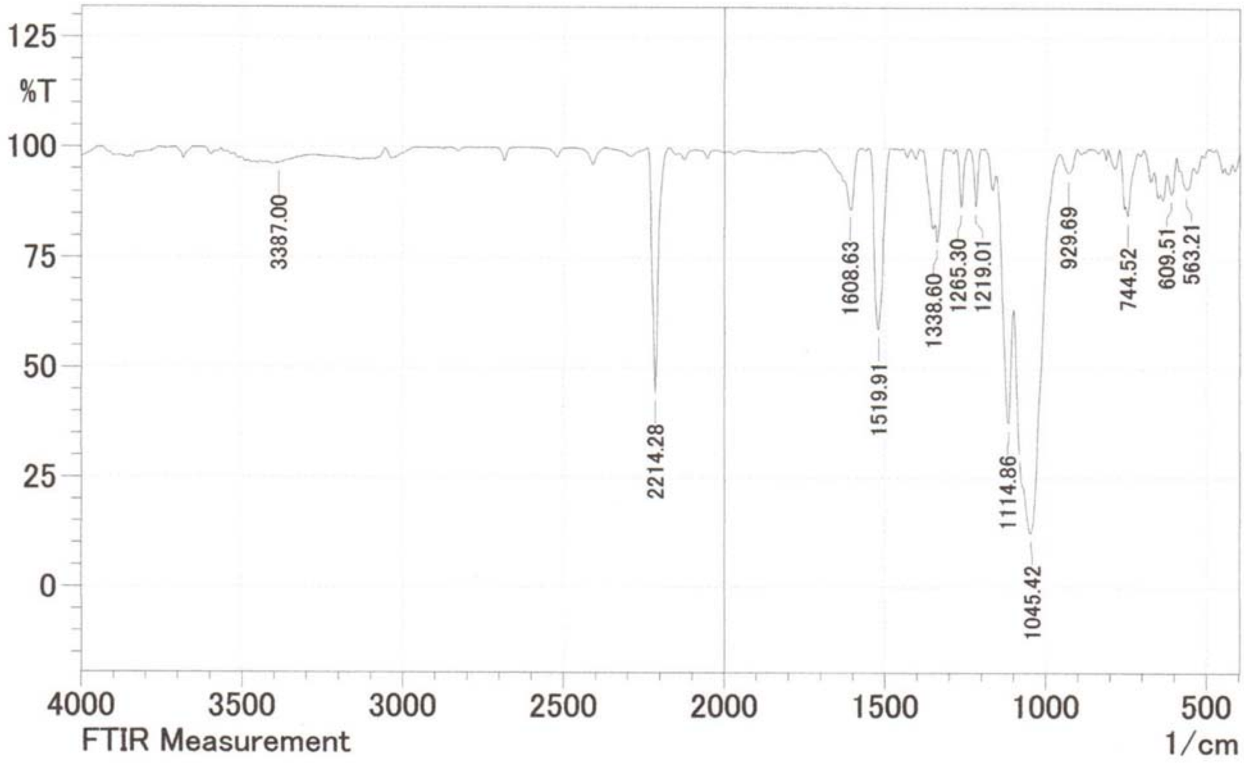

125
%T
100
75
50
25
0
3387.00
2214.28
1608.63
1519.91
1338.60
1265.30
1219.01
1114.86
1045.42
929.69
744.52
609.51
563.21
4000
3500
3000
2500
2000
1500
1000
500
FTIR Measurement
1/cm

**HRMS**

**Elemental Composition Report** **Page 1**

**Single Mass Analysis**

Tolerance = 50.0 mDa / DBE: min = -1.5, max = 100.0
Element prediction: Off
Number of isotope peaks used for i-FIT = 3

Monoisotopic Mass, Even Electron Ions
2 formula(e) evaluated with 1 results within limits (up to 50 best isotopic matches for each mass)
Elements Used:
C: 30-30 H: 16-16 N: 6-6 O: 2-2 F: 6-6 Na: 0-1 Cl: 1-1
20230422_4-1 13 (0.455)
1: TOF MS ES-

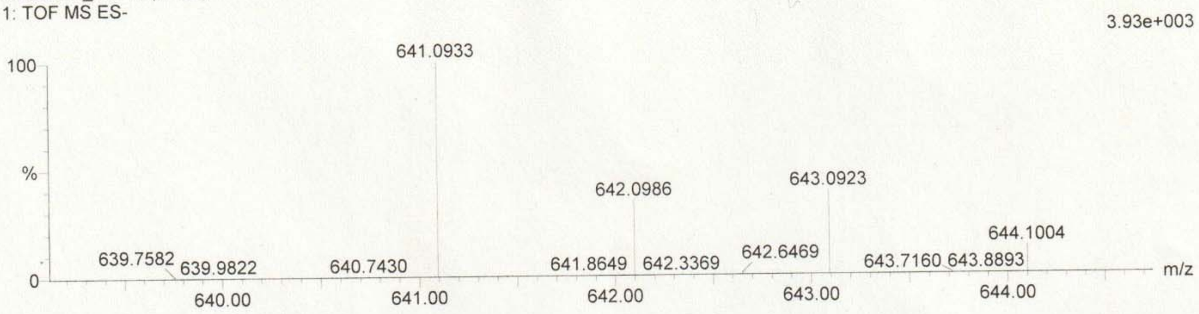


| | | | | | | | |
|---|---|---|---|---|---|---|---|
| Minimum: | | | | -1.5 | | | |
| Maximum: | | 50.0 | 80.0 | 100.0 | | | |
| Mass | Calc. Mass | mDa | PPM | DBE | i-FIT | i-FIT (Norm) | Formula |
| 641.0933 | 641.0927 | 0.6 | 0.9 | 22.5 | 32.8 | 0.0 | C30 H16 N6 O2 F6 Cl |

**1, 2, 3, 3-tetramethyl-3*H*-indol-1-ium iodide (6) [3]**

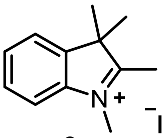

6

**$^{1}$H NMR**

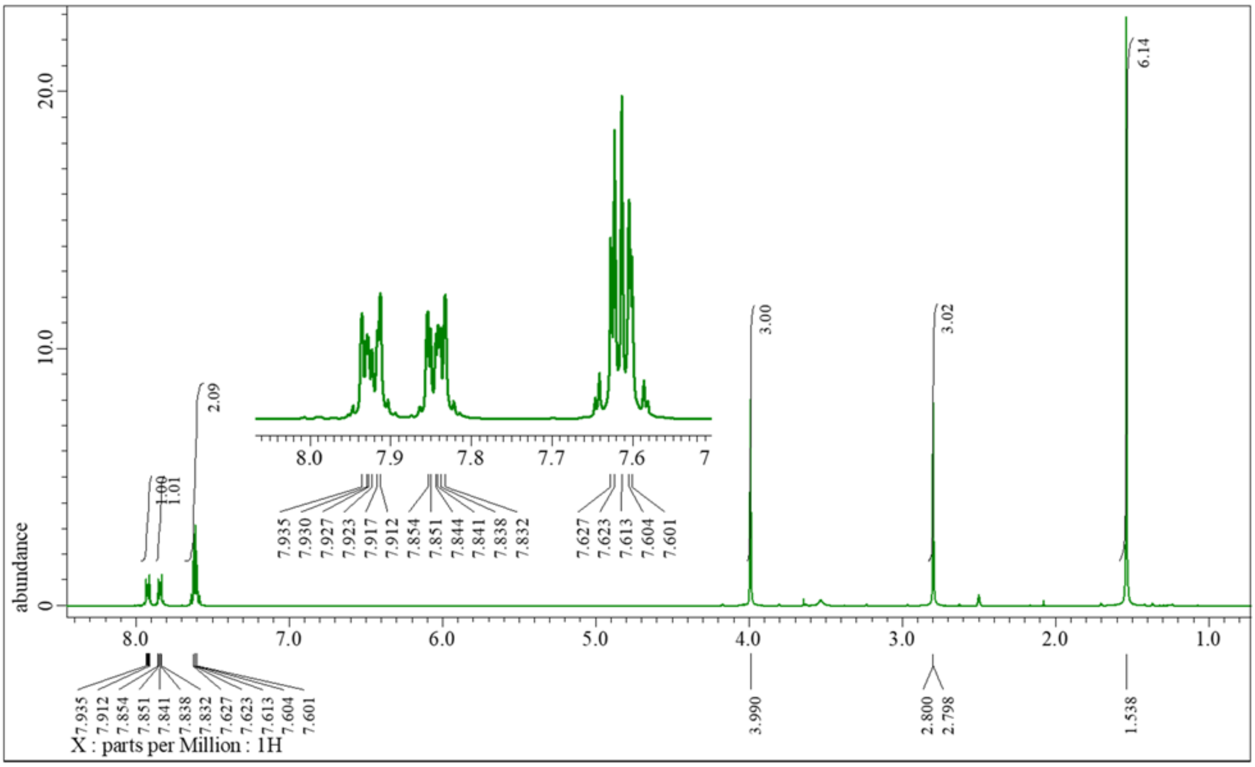


**$^{13}$C NMR**

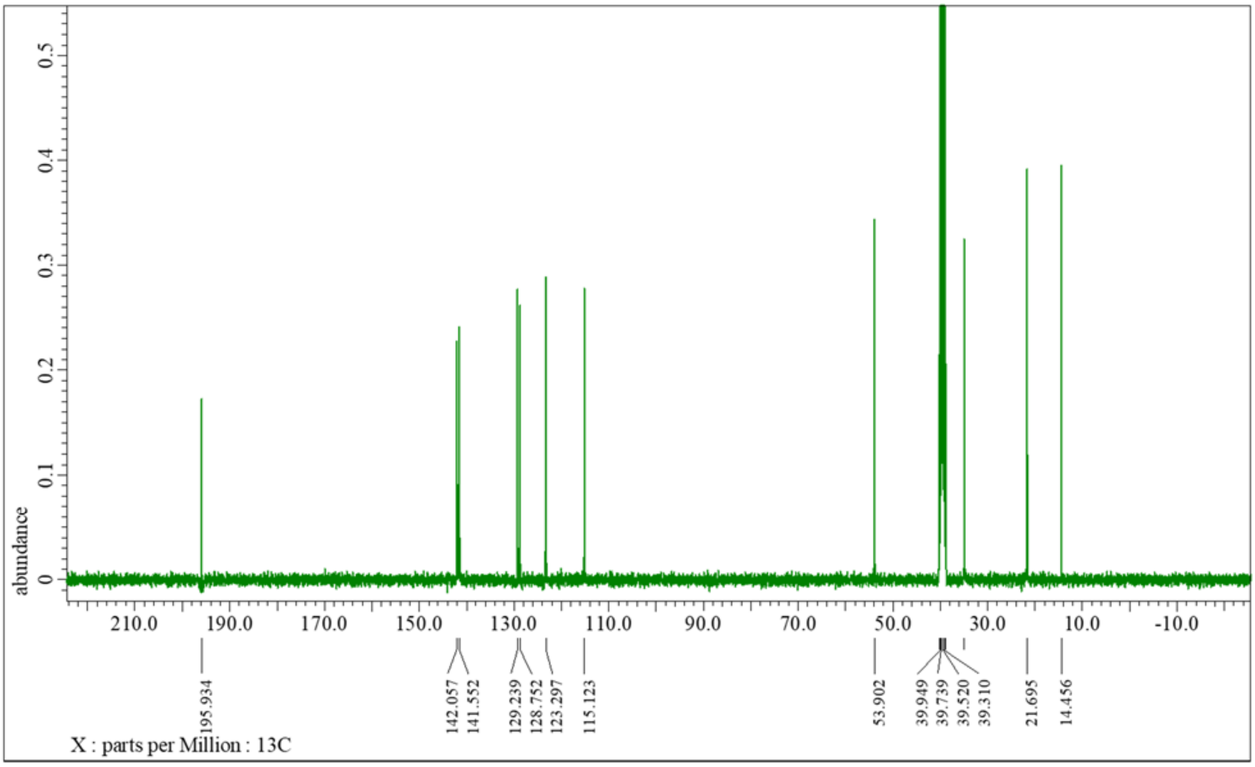

**IR**

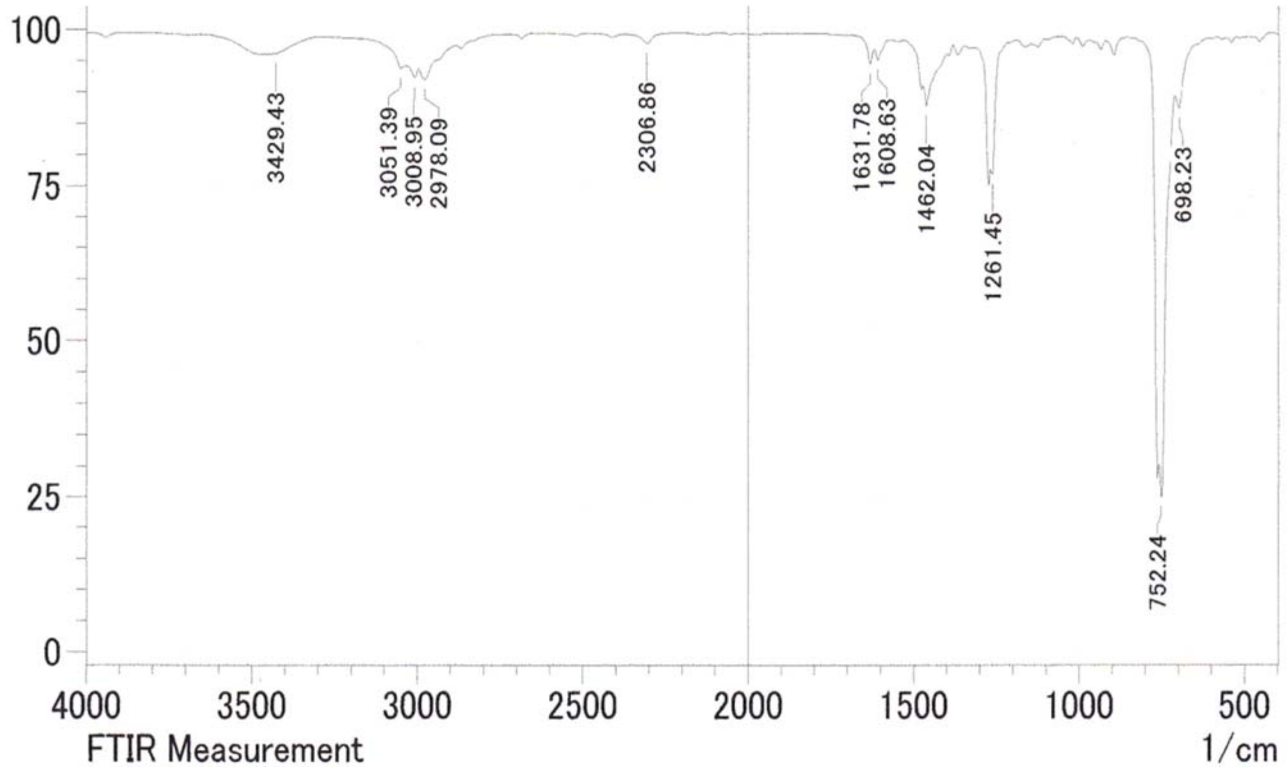


**HRMS**

Monoisotopic Mass, Even Electron Ions
5 formula(e) evaluated with 1 results within limits (up to 50 closest results for each mass)
Elements Used:
C: 12-12 H: 16-16 N: 1-1 Na: 0-1 Se: 0-2
20211220_7 7 (0.250)
1: TOF MS ES+

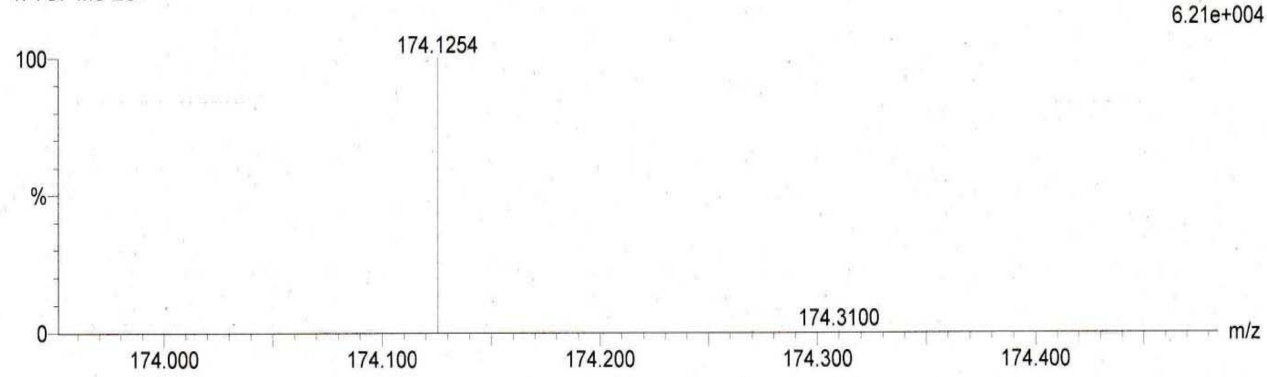


| | | | | | | | |
|---|---|---|---|---|---|---|---|
| Minimum: | | | | -1.5 | | | |
| Maximum: | | 1000.0 | 10.0 | 50.0 | | | |

| Mass | Calc. Mass | mDa | PPM | DBE | i-FIT | i-FIT (Norm) | Formula |
|---|---|---|---|---|---|---|---|
| 174.1254 | 174.1283 | -2.9 | -16.7 | 5.5 | 42.7 | 0.0 | C12 H16 N |

**2-((*E*)-2-((*E*)-2-chloro-3-(2-((*E*)-1,3,3-trimethylindolin-2-ylidene)ethylidene)cyclohex-1-en-1-yl)vinyl)-1,3,3-trimethyl-3*H*-indol-1-ium iodide (7). [4]**

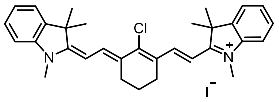


7

**$^1$H NMR**

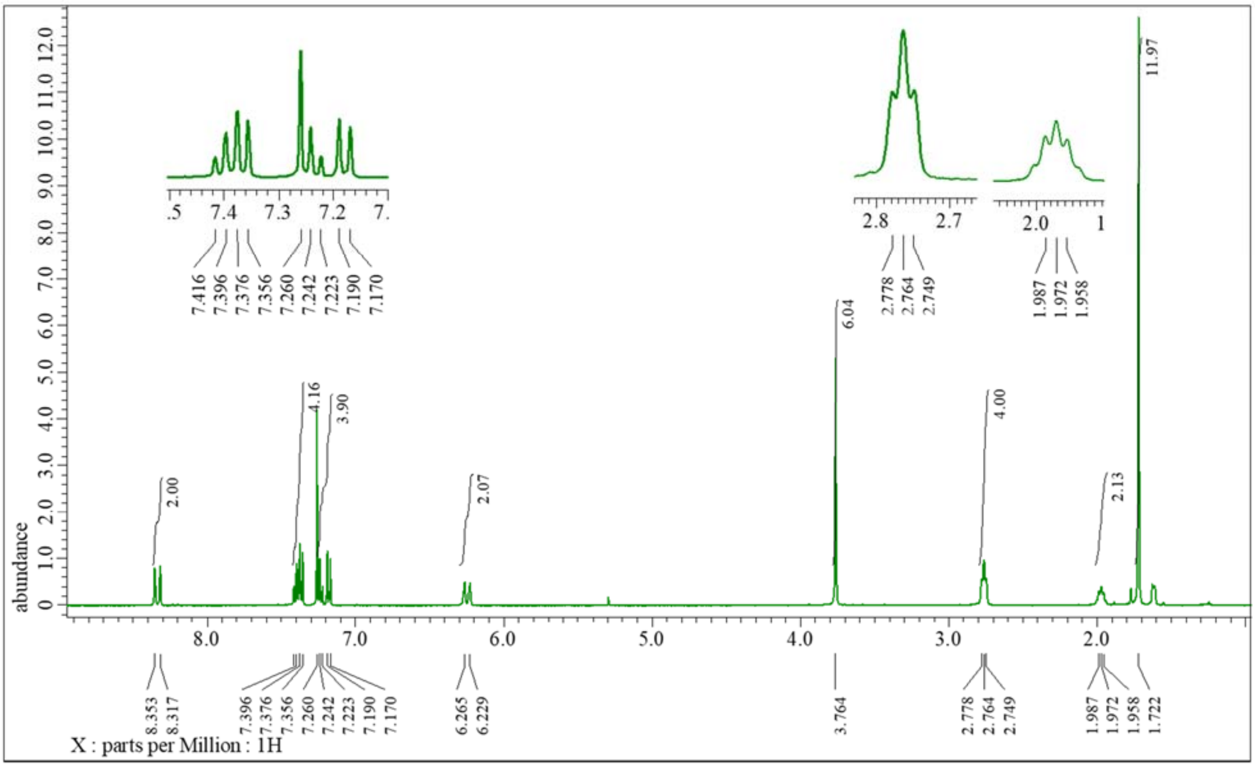


**$^{13}$C NMR**

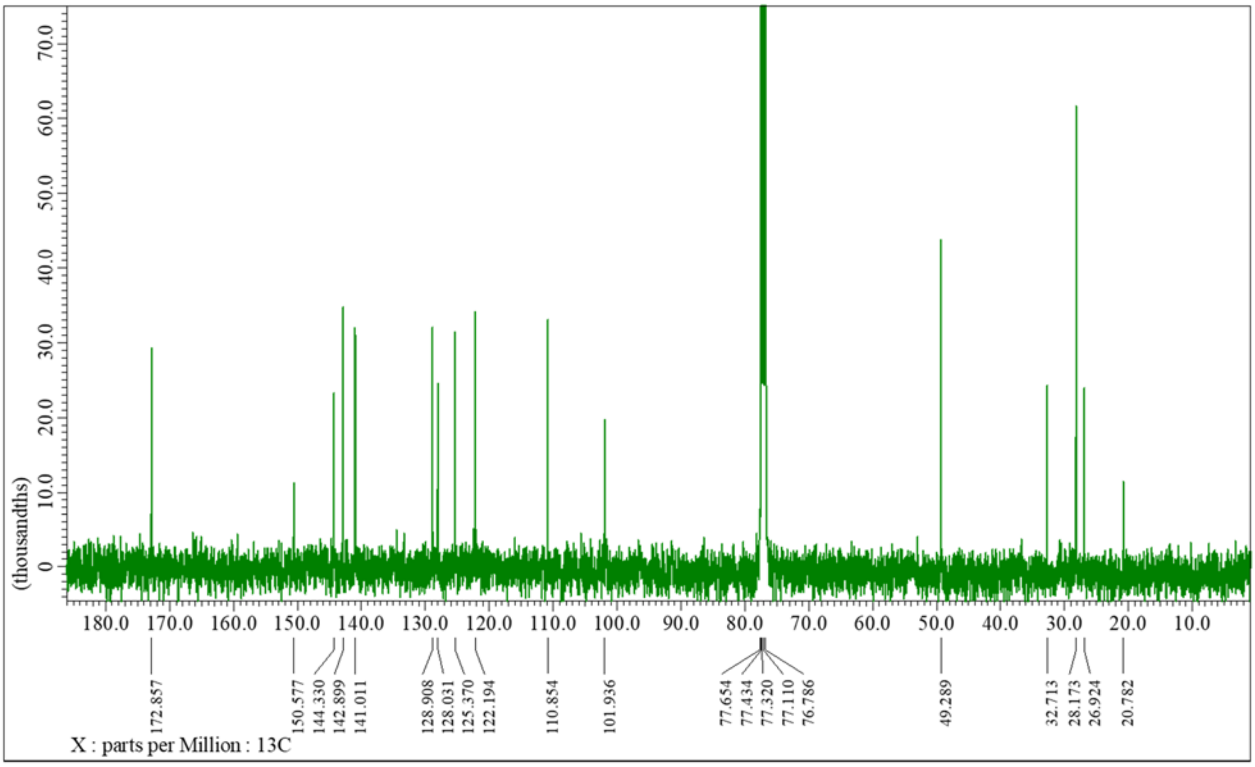

**IR**

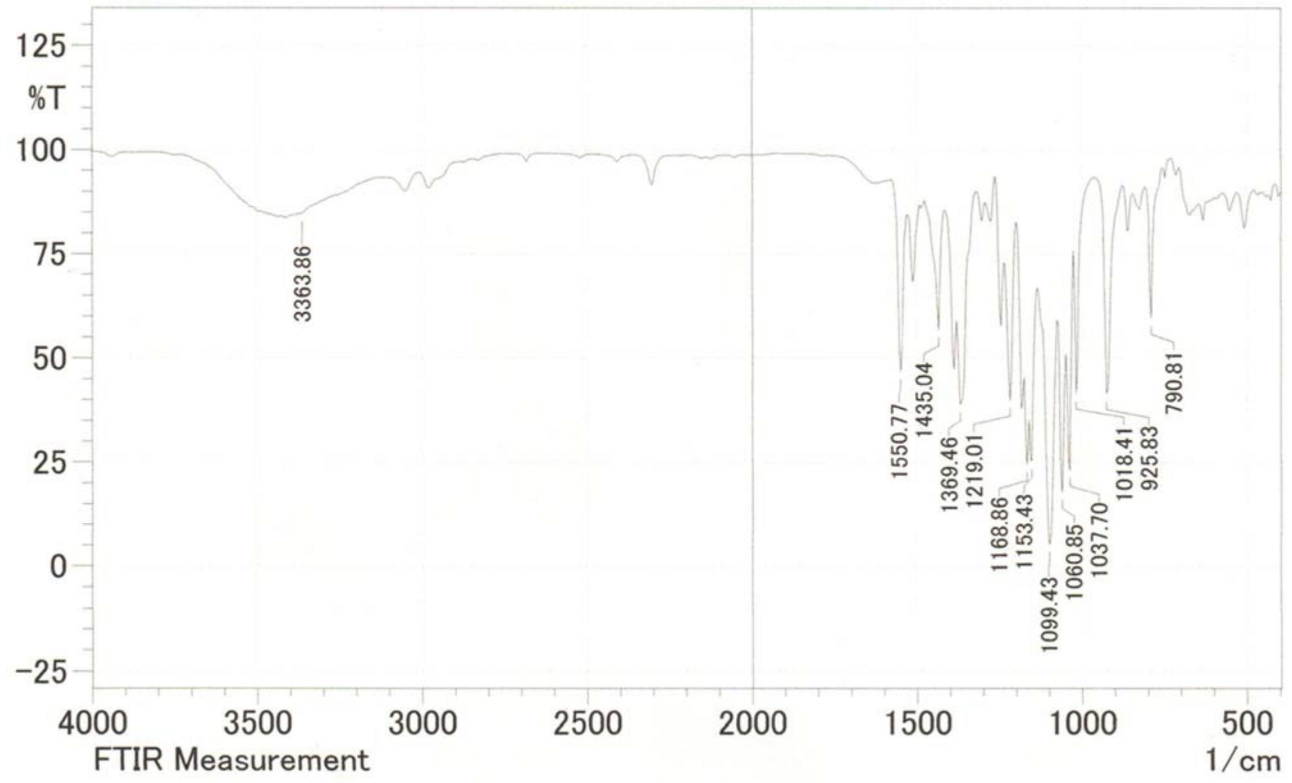


**HRMS**

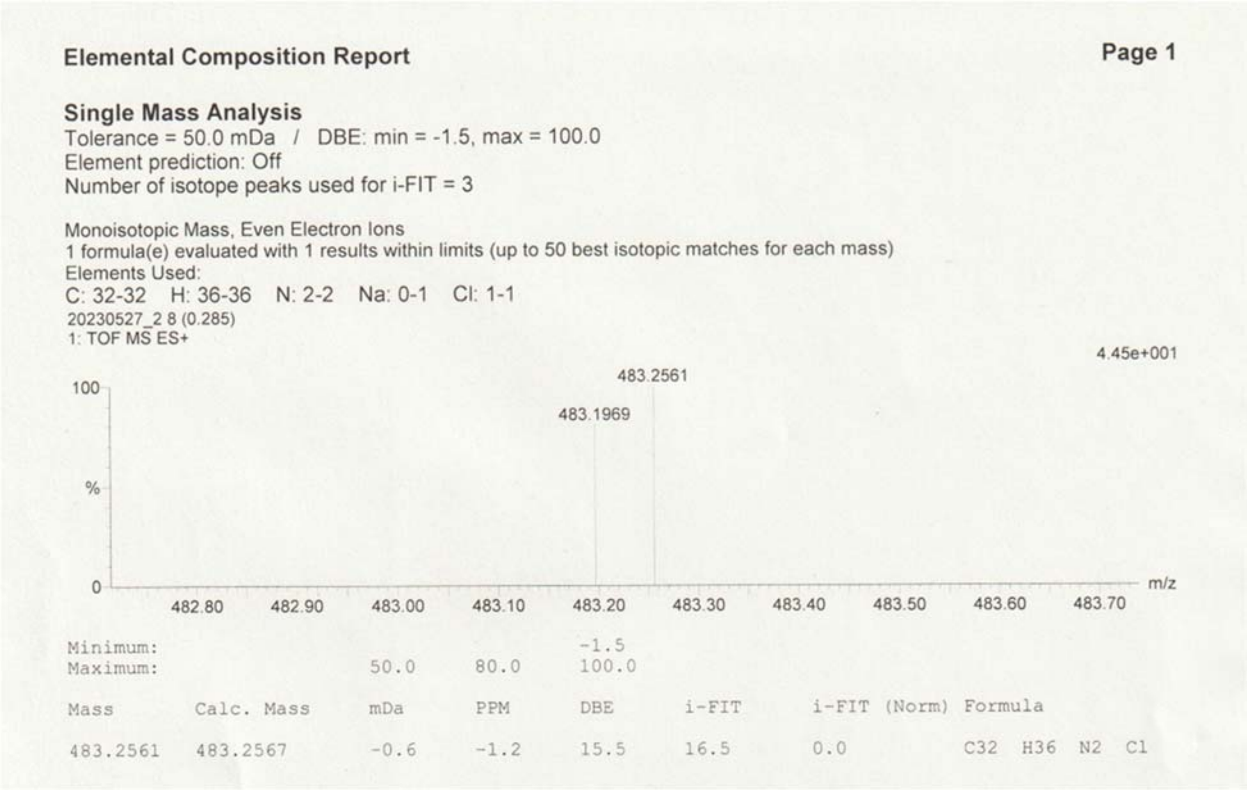

**Elemental Composition Report** **Page 1**

**Single Mass Analysis**
Tolerance = 50.0 mDa / DBE: min = -1.5, max = 100.0
Element prediction: Off
Number of isotope peaks used for i-FIT = 3

Monoisotopic Mass, Even Electron Ions
1 formula(e) evaluated with 1 results within limits (up to 50 best isotopic matches for each mass)
Elements Used:
C: 32-32 H: 36-36 N: 2-2 Na: 0-1 Cl: 1-1
20230527_2 8 (0.285)
1: TOF MS ES+



| | | | | | | | |
|---|---|---|---|---|---|---|---|
| Minimum: | | | | -1.5 | | | |
| Maximum: | | 50.0 | 80.0 | 100.0 | | | |
| Mass | Calc. Mass | mDa | PPM | DBE | i-FIT | i-FIT (Norm) | Formula |
| 483.2561 | 483.2567 | -0.6 | -1.2 | 15.5 | 16.5 | 0.0 | C32 H36 N2 Cl |

**2-((*E*)-2-((*E*)-2-Chloro-3-(2-((*E*)-1,3,3-trimethylindolin-2-ylidene)ethylidene)cyclohex-1-en-1-yl)vinyl)-1,3,3-trimethyl-3H-indol-1-ium ((*Z*)-4-((*E*)-2-(2-chloro-3-((*E*)-2-(4-cyano-5-(dicyanomethylene)-2-methyl-2-(trifluoromethyl)-2,5-dihydrofuran-3-yl)vinyl)cyclohex-2-en-1-ylidene)ethylidene)-3-cyano-5-methyl-5-(trifluoromethyl)-4,5-dihydrofuran-2-yl)dicyanomethanide (8a)**

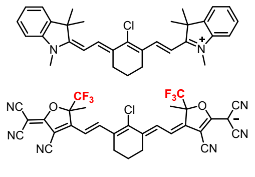

8a

**$^{1}$H NMR**

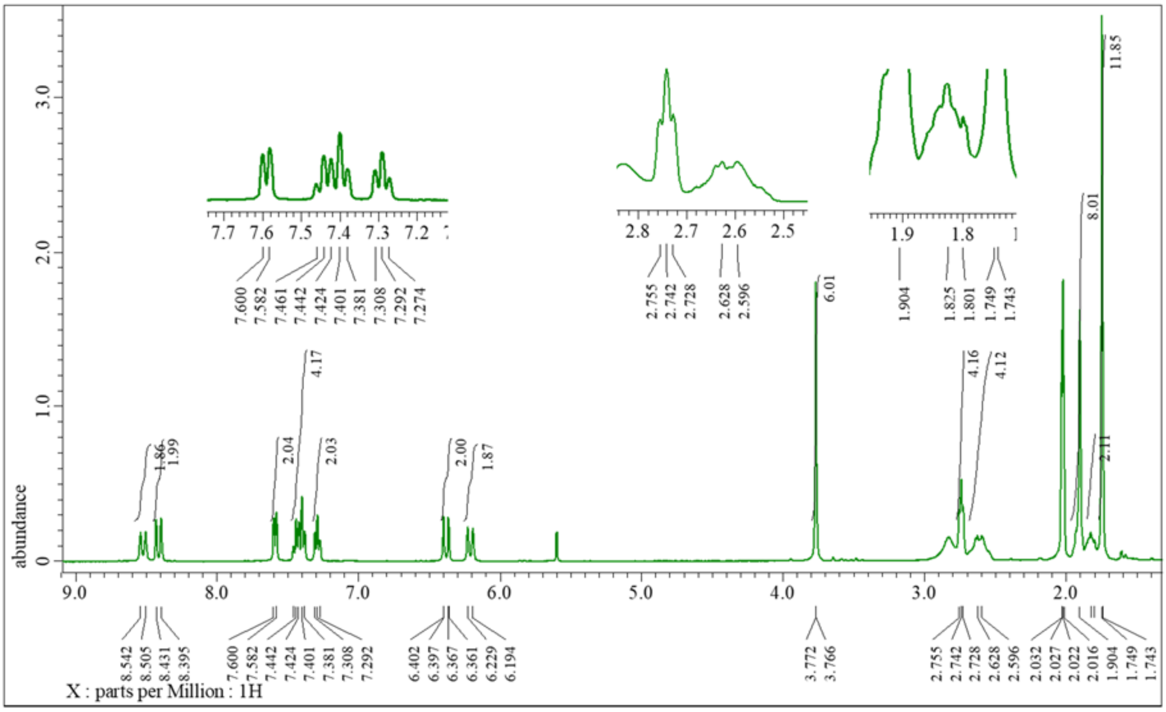

**$^{13}$C NMR**

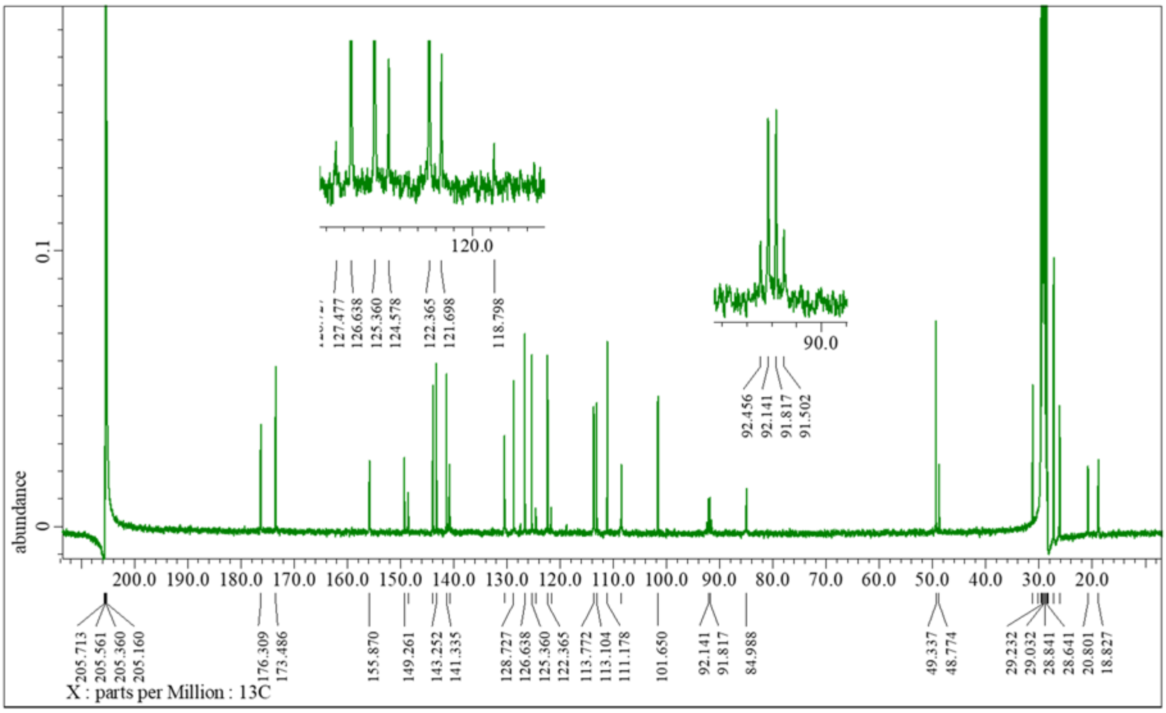

## $^{19}$F NMR

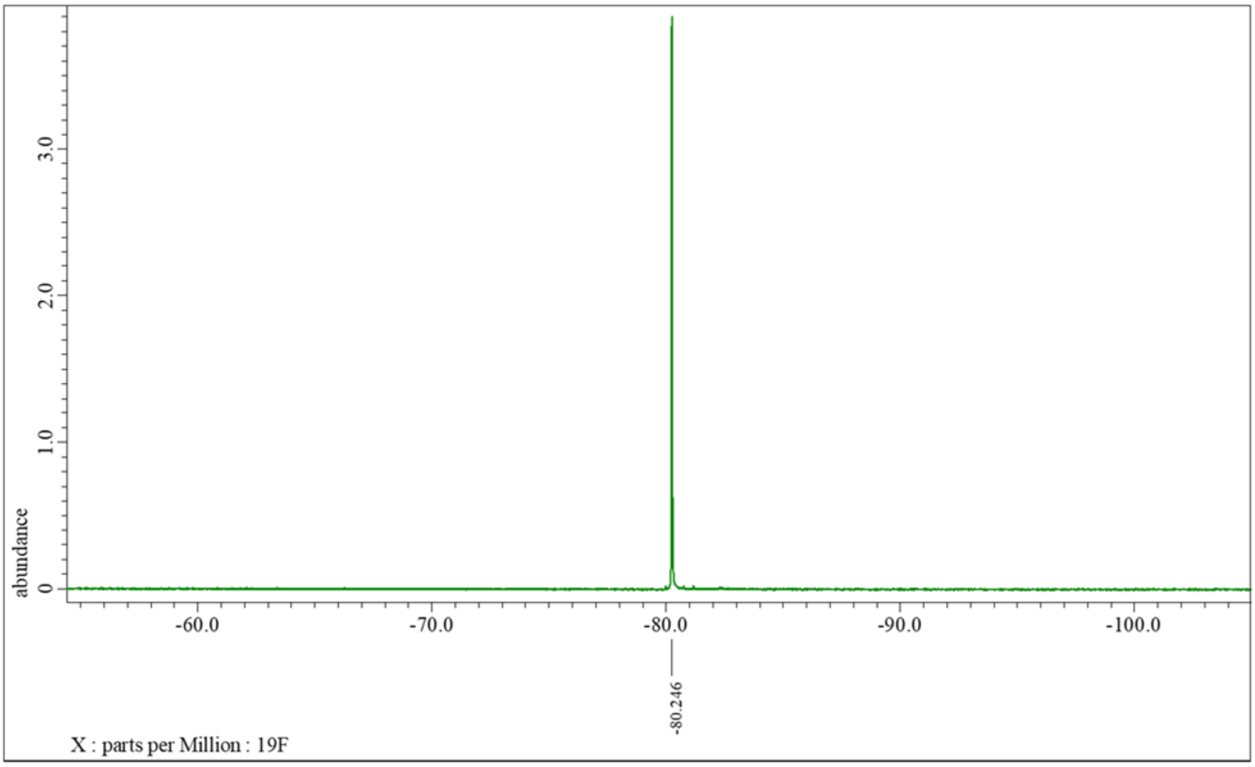

abundance
3.0
2.0
1.0
0
-60.0
-70.0
-80.0
-90.0
-100.0
-80.246
X : parts per Million : 19F


## IR

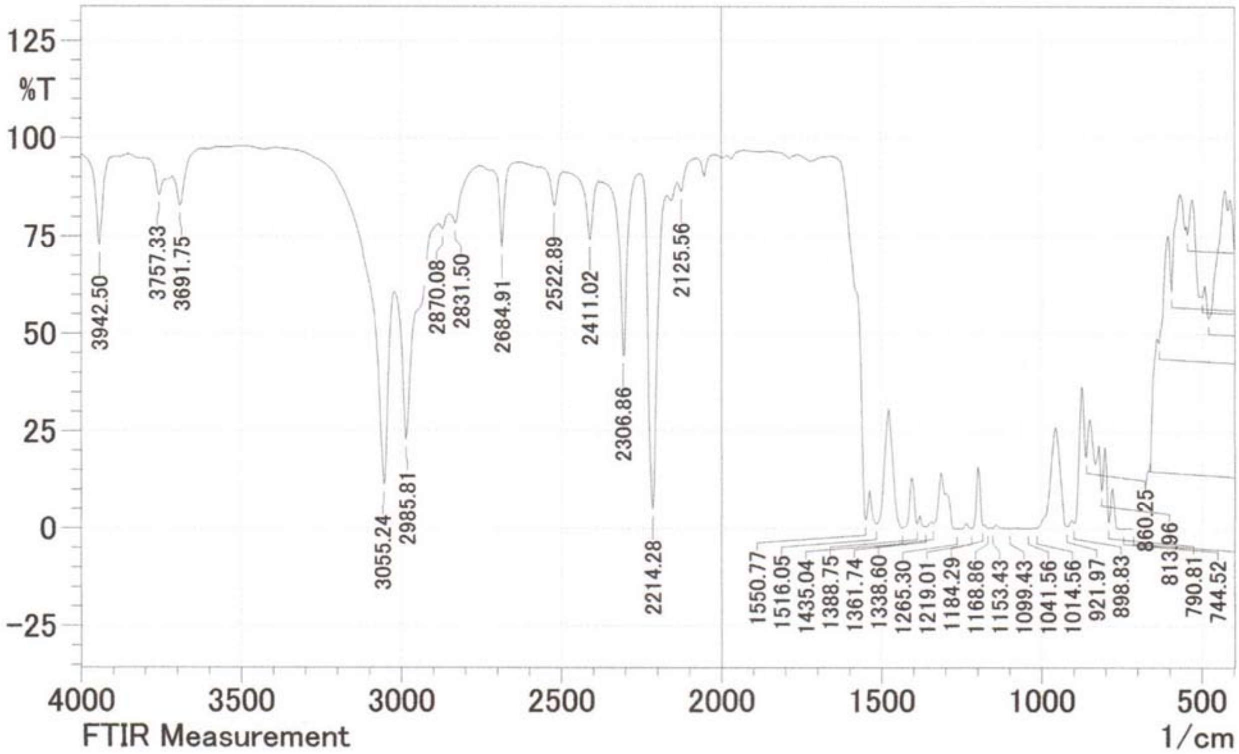

125
%T
100
75
50
25
0
−25
3942.50
3757.33
3691.75
3055.24
2985.81
2870.08
2831.50
2684.91
2522.89
2411.02
2306.86
2214.28
2125.56
1550.77
1516.05
1435.04
1388.75
1361.74
1338.60
1265.30
1219.01
1184.29
1168.86
1153.43
1099.43
1041.56
1014.56
921.97
898.83
860.25
813.96
790.81
744.52
4000
3500
3000
2500
2000
1500
1000
500
FTIR Measurement
1/cm

**HRMS**

**Elemental Composition Report** **Page 1**

**Single Mass Analysis**
Tolerance = 30.0 mDa / DBE: min = -1.5, max = 50.0
Element prediction: Off
Number of isotope peaks used for i-FIT = 3

Monoisotopic Mass, Even Electron Ions
1 formula(e) evaluated with 1 results within limits (up to 50 best isotopic matches for each mass)
Elements Used:
C: 32-32 H: 36-36 N: 2-2 Na: 0-1 Cl: 1-1
20230422_6 23 (0.808)
1: TOF MS ES+ 3.01e+001

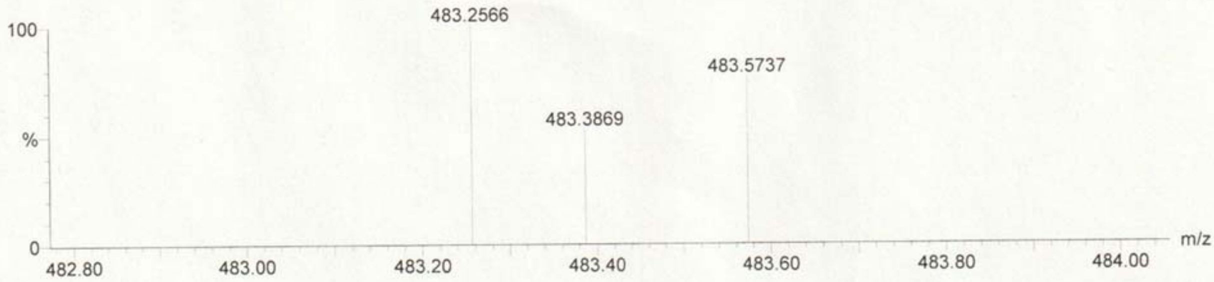


| | | | | | | | |
|---|---|---|---|---|---|---|---|
| Minimum: | | | | -1.5 | | | |
| Maximum: | | 30.0 | 80.0 | 50.0 | | | |
| Mass | Calc. Mass | mDa | PPM | DBE | i-FIT | i-FIT (Norm) | Formula |
| 483.2566 | 483.2567 | -0.1 | -0.2 | 15.5 | 27.4 | 0.0 | C32 H36 N2 Cl |

**Elemental Composition Report** **Page 1**

**Single Mass Analysis**
Tolerance = 50.0 mDa / DBE: min = -1.5, max = 100.0
Element prediction: Off
Number of isotope peaks used for i-FIT = 3

Monoisotopic Mass, Even Electron Ions
2 formula(e) evaluated with 1 results within limits (up to 50 best isotopic matches for each mass)
Elements Used:
C: 30-30 H: 16-16 N: 6-6 O: 2-2 F: 6-6 Na: 0-1 Cl: 1-1
20230422_5-1 8 (0.284)
1: TOF MS ES- 5.62e+004

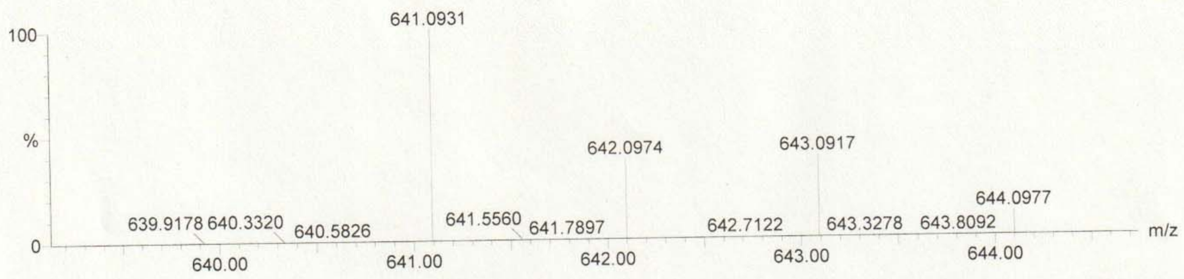


| | | | | | | | |
|---|---|---|---|---|---|---|---|
| Minimum: | | | | -1.5 | | | |
| Maximum: | | 50.0 | 80.0 | 100.0 | | | |
| Mass | Calc. Mass | mDa | PPM | DBE | i-FIT | i-FIT (Norm) | Formula |
| 641.0931 | 641.0927 | 0.4 | 0.6 | 22.5 | 73.6 | 0.0 | C30 H16 N6 O2 F6 Cl |

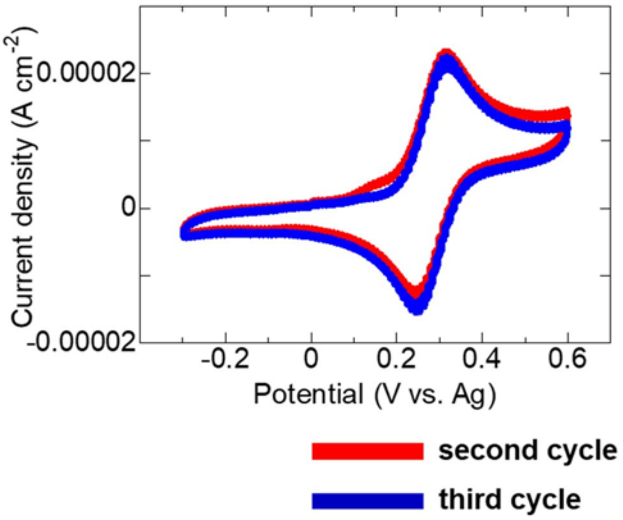


**Figure S1.** Cyclic voltammograms of the prepared anionic HMC dyes **5a** ($1.0 \times 10^{-3}$ M) in MeCN containing $Bu_4NClO_4$ (0.1 M) as the supporting electrolyte; scan rate of 200 mV $s^{-1}$

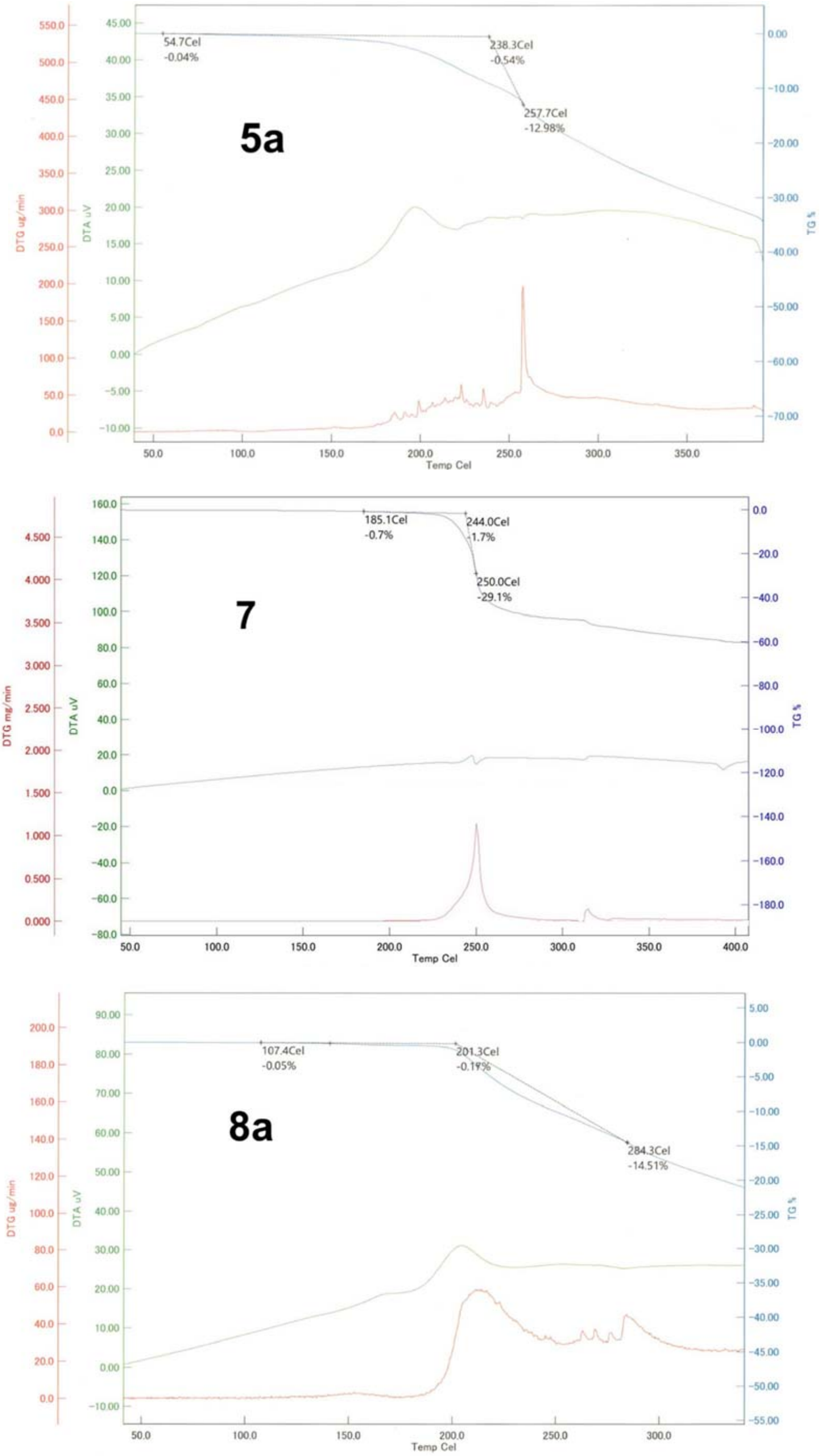


**Figure S2.** Results of TG-DTA measurements for **5a** and **7a**

## References

1. Liu, S.; Haller, M.A.; Ma, H.; Dalton, L.R.; Jang, S.-H.; Jen, A. K.-Y. Focused Microwave-Assisted Synthesis of 2,5-Dihydrofuran Derivatives as Electron Acceptors for Highly Efficient Nonlinear Optical Chromophores. *Adv. Mater.,* **2003**, *15*, 603–607.
2. Mukherjee, A.; Saha, P. C.; Das, R. S.; Bera, T.; Guha, S. Acidic pH-Activatable Visible to Near-Infrared Switchable Ratiometric Fluorescent Probe for Live-Cell Lysosome Targeted Imaging. *ACS Sens*., **2021**, *6,* 2141–2146.
3. Burdette, M. K.; Jenkins R.; Bandera, Y. P.; Jones, H.; Foulger, I. K.; Dickey, A.; Nieminen, A-L.; Foulger, S. H. Click-Engineered, Bioresponsive, and Versatile Particle–Protein–Dye System. *ACS Appl. Bio Mater*., **2019**, *2, 8,* 3183– 3193.
4. Shibayama, M.; Uehashi, Y.; Ajioka, S.; Kubota, Y.; Inuzuka, T.; Funabiki, K. Vapochromism of indolenine-based heptamethine cyanine dye adsorbed on silica gel. *New J. Chem.*, **2023**, *47*, 5262- 5269.
